# Probiotic potential and safety properties of *Limosilactobacillus fermentum* A51 with high exopolysaccharide production

**DOI:** 10.3389/fmicb.2025.1498352

**Published:** 2025-01-21

**Authors:** Guangqiang Wei, Daodian Wang, Teng Wang, Gao Wang, Yunmei Chai, Yufang Li, Minhui Mei, Hao Wang, Aixiang Huang

**Affiliations:** ^1^College of Food Science and Technology, Yunnan Agricultural University, Kunming, China; ^2^Sericulture and Apiculture Research Institute, Yunnan Academy of Agricultural Sciences, Mengzi, Yunnan, China; ^3^Heqing County Inspection and Testing Institute, Heqing, Yunnan, China

**Keywords:** *Limosilactobacillusfermentum* A51, whole genome sequencing, probiotic properties, phenotype analysis, safety

## Abstract

**Introduction:**

Exopolysaccharides (EPS) produced by Lactic acid bacteria have many health benefits and unique physicochemical properties. They are widely used in the food industry to improve viscosity, mouthfeel, and textural properties of foods. In our previous studies, *Limosilactobacillus fermentum* A51 (*L. fermentum* A51) isolated from yak yogurt exhibited high EPS production capacity and was applied to improve the texture of yogurt. In this study, whole genome sequencing analysis and corresponding *in vitro* assays were performed to investigate the probiotic potential and safety properties of *L. fermentum* A51.

**Results:**

Scanning electron microscopy (SEM) observed that *L. fermentum* strain A51 adhered into clusters and its colony exhibited the obvious silk drawing phenomenon. Whole genome mapping revealed that *L. fermentum* A51 genome is 2,188,538 bp, and with an average guanine and cytosine (GC) content of 51.28%. PGAAP annotation identified 2,152 protein-encoding genes and 58 rRNAs, 15 tRNAs, and 5 5sRNAs. Hemolysis and antibiotic resistance tests, combined with the analysis of genes involved in antibiotic resistance, virulence factor, and hemolysins, suggested that *L. fermentum* A51 is safe. Fifty-one carbohydrate active enzyme genes in the whole genome sequence of *L. fermentum* A51 were annotated by carbohydrate active enzymes (CAZymes). Furthermore, *L. fermentum* A51 possesses adhesion, acid tolerance, bile salt tolerance, and heat tolerance genes (*srtA*, *tuf*, *Bsh*, *nhaC, Ntn, cfa*), antioxidant (*nrfA, npr, nox2, tps*), antibacterial genes (*Idh* and *Dld*) EPS synthesis-related genes (*glf, epsG, gtf, Wzz, Wzx, Wzy*), and signal molecule A1-2 synthesis-related genes (*luxS*, *pfs*). These probiotic genes were verified by quantitative real-time PCR. *In vitro* assays confirmed that *L. fermentum* A51 showed good tolerance to simulated gastrointestinal tract (8.49 log CFU/mL), 0.3% bile salt (39.06%), and possessed adhesion (86.92%), antioxidant (70.60–89.71%), and antimicrobial activities, as well as EPS and signaling molecule AI-2 synthesis capacities.

**Conclusion:**

Collectively, our findings have confirmed that *L. fermentum* A51 is safe and exhibits good probiotic properties, thus recommending its potential application in the production of value-added fermented dairy products.

## 1 Introduction

LAB are generally recognized as safe (GRAS) microorganisms and are widely and safely used in the production of fermented dairy, fruit and vegetable products, and fermented meat products (Stefanovic et al., 2017; Saadat et al., 2019). Over the past decade, LAB have gained widespread attention for their health-promoting properties and are thus increasingly used as supplements in the food industry (Vasiee et al., 2018; Ma et al., 2019). The World Food Organisation (FAO) and the World Health Organisation (WHO) define probiotics as living microorganisms that produce health benefits for the host when consumed in sufficient quantities. *Limosilactobacillus fermentum* (*L. fermentum*) is one of the most versatile LAB strains. Studies have reported that *L. fermentum* exhibits probiotic activities such as antibacterial, antioxidant, anti-inflammatory, and immunomodulatory activities, in addition to the ability in prevention and treatment of hyperuricemia and gout, prevention of diseases and bile salt tolerance, among other functions (Zhao et al., 2019; Zhao et al., 2021a; Zhao et al., 2021b; Łepecka et al., 2023). The probiotic properties of *L. fermentum* are positively correlated with the functional metabolites that it produces. For instance, lactic acid and bacteriocin NQGPLGNAHR produced by *L. fermentum* show anti-adhesive and bactericidal activity against *Staphylococcus aureus* (Song et al., 2021). EPS produced by *L. fermentum* have many health benefits and unique physicochemical properties. They are widely used in the food industry to improve viscosity, mouthfeel, and textural properties of foods (Korcz and Varga, 2021). *L. fermentum* MWLf-4 and *L. fermentum* MWLp-12 have a high production of EPS, which can be used to increase the viscosity of fermented milk (Wang et al., 2023). A study reveals that *L. fermentum* U-21 possesses superior *in-vivo* and *in-vitro* antioxidant activity (Grishina et al., 2023). Notably, *L. fermentum* has been widely used in the food industry. In the recent past, studies have gradually extended from physiological and biochemical analyses to conducting further investigation of molecular mechanisms and genetic properties (Nordstrm et al., 2021).

LAB exhibits various probiotic activities; however, it is time-consuming and expensive to explore the health benefits of LAB through *in-vitro* and *in-vivo* experiments. Recently, the development of high-throughput genomic methods has provided the means for rapidly understanding the genetic and probiotic properties of LAB, including their genes, metabolic capabilities, and potential health benefits (Wang et al., 2023). For instance, Wang et al. (2023) identified *tagE* and *glmU* genes regulating EPS production in *L. fermentum* MWLf-4 and the *cps* gene cluster and *galE* gene in *L. plantarum* MWLp-12 using whole gene sequencing. Liu et al. (2022) identified potential genes involved in the probiotic functions of *L. plantarum* DMDL 9010, including stress responses, bile salt resistance, adhesion ability, EPS biosynthesis and plantaricin biosynthesis using complete genome sequencing. Gao et al. (2020) also employed whole genome sequencing to identify potential genes involved in the probiotic function of *L. plantarum* Y44, including stress-related genes (*groES-groEL* and *CLIC*), gastric and intestinal transit tolerance-related genes (*Bsh* and *cfa*), antioxidant activity-related genes (*nox, nrdH,* and *trxB*), and EPS synthesis-related genes (*glf*, *epsD*, *gtf*, *Wzx*, *Wzy,* and *wzx*). Whole genome sequencing results indicate that *L. plantarum* Y42 genome contains genes associated with LuxS/AI-2 quorum sensing (QS) system (Li et al., 2023). Meanwhile, whole genome sequencing has been used to rapidly analyze safety-related biological information of LAB, including antimicrobial resistance genes and virulence factor gene annotations (Li et al., 2023; Lu et al., 2023). Based on the above research, it is apparent that combining whole genome sequencing and phenotyping analyses can provide insights into the probiotic properties and safety of LAB (Gu et al., 2023).

The evaluation of the safety and probiotic properties of LABs is a prerequisite for their application in the food industry. In our previous study, *L. fermentum* A51 isolated from naturally fermented yak yogurt was proven to have good EPS production capacity (452.728 mg/L) and fermentation properties. Especially, *L. fermentum* A51 was successfully applied to improve the textural properties of yogurt (Wei et al., 2023). However, the safety and probiotic properties of *L. fermentum* A51 are still unknown. In this study, the whole genome of *L. fermentum* A51 was sequenced using the third-generation sequencing technique. The antibiotic sensitivity and hemolytic activity of *L. fermentum* A51 were determined. Furthermore, the probiotic activities and probiotic genes, including gastrointestinal digestive tolerance, adhesion, antioxidant, and antimicrobial activities, as well as EPS and signaling molecule AI-2 synthesis capacity were investigated. The results provide insights into the probiotic properties and safety of *L. fermentum* A51, and will guide its application in the production of value-added fermented dairy products with probiotic properties.

## 2 Materials and methods

### 2.1 Materials

#### 2.1.1 Bacterial strains

*L. fermentum* A51 (Gene bank accession number: CP132542) used in the current study was isolated from naturally fermented yak yogurt in Yunnan province and identified by Gram stain reaction, morphological, and 16S rDNA sequence analysis. Subsequently, sequence similarity was assessed using BLAST tool, and a phylogenetic tree was constructed using the neighbor-joining method in Mega 7.0 software according to 16S rRNA gene sequences from 17 Lactobacillus strains. The strain has been deposited in the China Center for Type Culture Collection (CCTCC NO: M 2023861). The strain was stored at the College of Food Science and Technology, Yunnan Agricultural University, Kunming, China. The bacterial strains were preserved at −80°C in glycerol supplemented with 20% (v/v) nutrient broth. *Lactobacillus casei* Zhang was obtained from the Inner Mongolia Agricultural University. *Vibrio harveyi* BB170 (ATCC BAA-1117), which was used as an indicator bacteria, was purchased from Guangdong Microbial Culture Collection Center (GDMCC). *Escherichia coli* CICC 10003 and *Listeria monocytogenes* were purchased from the China Center of Industrial Culture Collection (Beijing). *Staphylococcus aureus* ATCC25923 was purchased from the American Type Culture Collection.

Columbia agar medium supplemented with 7% sterile defibrinated sheep blood and de Man-Rogosa-Sharpe (MRS) medium were purchased from Solarbio Science & Technology Co., Ltd., Beijing, China. Pepsin (from porcine gastric mucosa, ≥ 250 U/mg), trypsin (from bovine pancreas, ≥ 10,000 U/mg), and bile salts were purchased from Sigma-Aldrich® (St. Louis, MO, USA). In addition, 1,1-Diphenyl-2-picrylhydrazyl radical (DPPH) and 2,2′-diazo-bis (3-ethylbenzothiazolin-6-sulfonic acid) diammonium salt (ABTS) were purchased from Shanghai Jingchun Biochemical Technology Co., Ltd. (Shanghai, China).

### 2.2 Morphology of strains

One milliliter of third-generation *L. fermentum* A51 was gradient diluted to 10^−6^ folds, spread on MRS solid medium (Beijing Solarbio Science & Technology Co., Ltd. Beijing, China) and then incubated at 37°C for 48 h. Lengths of single colonies that were sticky and had obvious filament pulling with a sterilized gun were recorded. The colonies of *L. fermentum* A51 were Gram-stained and observed under a microscope (E200, Nikon, Japan).

The morphology of *L. fermentum* A51 was observed using biological scanning electron microscopy. Briefly, cells were obtained by centrifugation at 8,000 rcf, 4°C for 10 min, then washed twice with phosphate buffer (0.1 M, pH 7.2). The cells were fixed with 2.5% glutaraldehyde for 12 h and washed three times with phosphate buffer. Next, gradient dehydration with ethanol was performed for 10 min each time, and bacteria were precipitated by centrifugation at 8,000 rcf, 4°C for 10 min. Tert-butanol was used instead of ethanol for centrifugation. The bacteria were diluted with phosphate buffer, dropwise freeze-dried on slides, and sputter-coated with gold (Wu et al., 2015). Finally, cells were observed using a regulus 8,100 biological SEM (Hitachi Ltd., Tokyo, Japan).

### 2.3 Genome sequencing and annotation

*L. fermentum* A51 was sequenced using Illumina NovaSeq 6000 and PacBio Sequel II platforms by Biomarker Technologies Co, LTD. (Beijing, China). At least 1 μg of each genomic DNA sample was used for Illumina sequencing to construct a sequence library. DNA samples were sheared into 400–500 bp fragments using a Covaris M220 Focused Acoustic Shearer. Illumina sequencing libraries were prepared from the sheared fragments. The prepared libraries were then used for paired-end Illumina sequencing (2 × 150 bp) on an Illumina NovaSeq 6,000 machine. For PacBio sequencing, SMRTbell library inserts (20 kb) were sequenced, and subreads shorter than 500 bp were removed. The PacBio sequences were error-corrected, binned, and then assembled using the Canu assembler (version 2.2).^1^ Pilon software (version 1.24^2^) was used for polishing assemblies from Illumina short reads to improve genome quality. The predicted gene sequences were translated and searched against the National Center for Biotechnology Information (NCBI) database.^3^ Infernal v1.1.3, tRNAscan-SE v2.0, CRT v1.2, and IslandPath-DIMOB v0.2 were used for the prediction of rRNA, tRNA, CRISPR sequence, and gene island, respectively. The non-redundant protein (Nr) database, the Gene Ontology (GO) database, the Kyoto encyclopedia of genes and genomes (KEGG) database, and the carbohydrate-active enzymes (CAZy) database were used for annotation. Putative virulence genes were identified by comparing the whole genome of *L. fermentum* A51 with Virulence Factors of Pathogenic Bacteria (VFDB) database.^4^ Antibiotic resistance genes of *L. fermentum* A51 were identified using Comprehensive Antibiotic Research (CARD) database^5^ (Liu et al., 2022).

### 2.4 Detection of antibiotic sensitivity

The antibiotic sensitivity of *L. fermentum* A51 was evaluated using the disk diffusion method (Lu et al., 2023). Briefly, an MRS agar plate was overlaid with *L. fermentum* A51 culture (200 μL) containing 10^8^ CFU/mL and antibiotic discs containing erythromycin, chloromycetin, tetracycline, ciprofloxacin, clindamycin, ampicillin, gentamicin, kanamycin, vancomycin, and ofloxacin were placed on the plates under sterile conditions. After incubation at 37°C for 48 h, the diameter (mm) of the inhibition zones was measured and the level of antibiotic sensitivity of the strain was determined.

### 2.5 Hemolytic activity

The hemolytic activity of *L. fermentum* A51 was measured as described by Li et al. (2023). Briefly, *L. fermentum* A51 was inoculated on Columbia agar medium supplemented with 7% sheep blood and cultured anaerobically at 37°C for 48 h. *Lactobacillus casei* Zhang was used as the control strain. Hemolytic rings were observed to confirm the hemolytic activity of the strains. A transparent area meant *β*-hemolysis and has hemolysis, while the absence of a transparent zone (*γ*-hemolysis) or a green zone (*α*-hemolysis) meant non-hemolysis strain.

### 2.6 Probiotic properties evaluation of *Limosilactobacillus fermentum* A51

#### 2.6.1 Bile salt tolerance test

The bile salt tolerance of *L. fermentum* A51 was determined according to the method reported by Yan et al. (2023). The bacterial solution (1.0 mL, 10^9^ CFU/mL) was added to 9 mL MRS medium (with 0.1, 0.2, 0.3 and 0.4% w/v bile salt) and incubated at 37°C for 4 h. After the incubation, 100 μL of the fermentation broth was taken separately, and the number of viable bacteria was counted after appropriate dilution on MRS agar plates. The medium without added bile salts was used as a control and the survival rate was calculated.

#### 2.6.2 Gastrointestinal fluid tolerance test

The tolerance of *L. fermentum* A51 in *in vitro* simulated gastrointestinal was investigated according to the method reported by Fei et al. (2018) with slight modifications. The gastric juice (3 mg/mL pepsin) was prepared in phosphate buffered saline (PBS, pH 3.0). The intestinal fluid (0.1 mg/mL trypsin, 0.15% (w/v) bile salts) was prepared in PBS (pH 6.8). Activated bacterial solution was added to the artificial gastric juice and incubated at 37°C for 2 h before plate colony counting. After incubating in the simulated gastric fluid for 2 h at 37°C, 1 mL of activated bacterial solution was added to 9 mL of simulated artificial intestinal fluid (pH 8.0). After incubating at 37°C for 2 h, the intestinal fluid tolerance was determined by counting total viable cells. Distilled water was used as a blank control. *Lactobacillus casei* Zhang was used as the control strain.

#### 2.6.3 Detection of adhesion capacity

The adhesion capacity of *L. fermentum* A51 was assessed by hydrophobicity and self-aggregation tests (Peng et al., 2023). *Lactobacillus casei* Zhang was used as the control strain. Briefly, the washed bacterial sludge was taken, and the concentration of bacterial suspension was adjusted to 10^6^–10^7^ CFU/mL with saline; the absorbance at 600 nm was measured and counted as A_0_. Then 1 mL each of xylene, chloroform, and ethyl acetate were added to 3 mL of bacterial suspension and mixed well. The aqueous phase was taken after 20 min of standing and its absorbance at 600 nm was measured and counted as A_1_. The hydrophobicity of the strain to the solvent was calculated according to the following formula:


$$\mathrm{Hydrophobicity}\;\left(\%\right)=\left(1-\frac{A_{1}}{A_{0}}\right)\times 100\%$$

A total of 5 mL of the bacterial suspension was pipetted into an EP tube, vortexed for 30 s, and then left to stand for 5 h. The supernatant was aspirated at 1 h intervals and the absorbance value at 600 nm was determined. The auto aggregation capability of the strain was calculated according to the following formula:


$$\mathrm{Aggregation capability}\;\left(\%\right)=\left(1-\frac{A_{t}}{A_{0}}\right)\times 100\%$$

where At represents the absorbance value of the bacterial suspension at 8, 12, and 24 h and A0 represents the absorbance value at 0 h.

#### 2.6.4 Assays for antioxidant activities

The radical scavenging activity of *L. fermentum* A51 against DPPH radical, ABTS radical, and reducing capability were determined following previous methods (Sun et al., 2022; Liu et al., 2021). *Lactobacillus casei* Zhang was used as the control strain.

#### 2.6.5 Evaluation of antibacterial activity

The antimicrobial activity of *L. fermentum* A51 against *Escherichia coli* (*E. coli*), *Staphylococcus aureus* (*S. aureus*) and *Listeria monocytogenes* (*L. monocytogenes*) was determined by oxford cup assay (Delgado et al., 2007). Briefly, the *E. coli, S. aureus* and *L. monocytogenes* cultured to a mid-log phase were mixed with LB agar broth at 37°C and then poured into Petri dishes. Then, 150 μL of *L. fermentum* A51 cell-free supernatant (CFS) was added to an 8-mm LB agar plate. The plates were then incubated at 37°C and then checked for inhibition after 12 h. *Lactobacillus casei* Zhang was used as the control strain.

#### 2.6.6 Determination of EPS production

The EPS synthesis ability of *L. fermentum* A51 was measured according to Li M. et al. (2022). Briefly, *L. fermentum* A51 was inoculated at 5% (V/V) in fresh MRS medium and incubated at 37°C for 0, 2, 4, 6, 8, 10, 12, 14, 16, 18, 20, 22, 24, and 26 h. The fermentation broth was centrifuged at 6250 rcf for 15 min at 4°C to obtain CFS. Then, trichloroacetic acid (TCA) at a final concentration of 4% was added to the CFS to remove proteins. The solution was centrifuged at 8,000 rcf for 15 min at 4°C to obtain supernatant. The supernatant was concentrated to one-third of its original volume; and after 99.95% chilled ethanol at three times its volume was added, it was stored overnight at 4°C. The precipitate was collected and redissolved in deionized water. After dialysis and lyophilization, EPS produced by *L. fermentum* A51 was obtained, and the EPS content was determined by phenol-sulfuric acid method.

#### 2.6.7 Determination of activity of signaling molecule AI-2

The ability of *L. fermentum* A51 to secrete the signaling molecule AI-2 was determined based on the method of Ma et al. (2022). *Vibrio harveyi* (*V. harveyi*) BB170 was cultured overnight in AB medium until the OD_600_ reached 0.9–1.1. The *V. harveyi* BB170 solution was diluted with fresh autoinducer bioassay (AB) medium at a volume ratio of 1:100. Sterile supernatants (obtained by filtering through a 0.22 μm microbial filter) of *L. fermentum* A51 incubated at 37°C for 0, 2, 4, 6, 8, 10, 12, 14, 16, 18, 20, 22, 24, and 26 h, negative control and medium control were mixed with the diluted *V. harveyi* BB170 solution at a volume ratio of 1:50 and incubated at 100 rpm and 30°C in a shaker (Shanghai Zhichu Instrument Co., Ltd. Shanghai, China). The AI-2 content of each test group was calculated when the fluorescence value of the negative control is the lowes. The content of AI-2 is the ratio of the fluorescence value of the experimental group to the fluorescence value of the medium group.


$$\begin{array}{l} \mathrm{Relative}\;\mathrm{fluorescence}\;\mathrm{intensity}\;\mathrm{of}\;\mathrm{the}\;\mathrm{sample}=\\ \frac{\mathrm{Fluorescence}\;\mathrm{intensity}\;\mathrm{value}\;\mathrm{of}\;\mathrm{the}\;\mathrm{sample}}{\mathrm{Fluorescence}\;\mathrm{intensity}\;\mathrm{value}\;\mathrm{of}\;\mathrm{the}\;\mathrm{medium}\;\mathrm{control}} \end{array}$$

### 2.7 Determination of expression levels of probiotic genes

Total RNA was isolated from *L. fermentum* A51 (incubated for 20 h) using RNA Extraction Kit (Beijing Tsingke Biotech Co., Ltd.). The A260/280 ratios of the extracted RNA were about 2.1. The total RNA was reverse-transcribed into cDNA using Goldenstar RT6 cDNA Synthesis Kit Ver 2 (Qingke Biotechnology [Beijing] Co., Ltd., China). Real-time PCR analysis of candidate genes, including *luxS*, *glf*, *epsG*, *gtf*, *Cfa*, *Wzz*, *Wzx*, *bsh*, *nhaC*, *npr*, *nox2, Idh*, and *Dld*, was performed using ABI, QuantStudioTM 1 Plus real-time fluorescence quantitative PCR instrument (Thermo Fisher, USA). Gene-specific primers used in the experiment are listed in Table 1. *Cfa* gene was used as an internal reference gene. Probiotic genes were analyzed using the 2^-ΔΔCt^ method.

**Table 1:** Sequences of primers used for real-time quantitative PCR.

Name	Sequence (5′–3′)	Size (bp)
*luxS*-F	AGAAATCGCCTACCACACCG	102
*luxS*-R	GACCATTCCTTGGCGGAGAA	
*glf*-F	CCAAGAAATGGCCGGCAAGA	157
*glf*-R	TGGCAGGCGACGAATAATGA	
*epsG*-F	AGTAACGAATGAGCGTCAAGAGA	184
*epsG*-R	CATCGCACCTGTCTTGCCTA	
*gtf*-F	CGTCGATGATGGATCAACTGATAA	109
*gtf*-R	GGCAGCACTAACTCCACCAT	
*Cfa*-F	CTACCAACGGACCCTGGAAC	144
*Cfa*-R	TCGATGTTGCCGGACTCAAA	
*Wzz*-F	ACGGTAAGCACCCAAACCAA	193
*Wzz*-R	TCTTGTTCGGGAAGGACTGG	
*Wzx*-F	TGTTGTTGATGCCGCAAAGA	154
*Wzx*-R	GATTAACCCAACTGCCACCC	
*bsh*-F	CAATGGACTGGGACGACCTC	122
*bsh*-R	GATCGAGCCACCACCAATGA	
*nhaC*-F	CATCGTTTGCACCCTCGTTG	111
*nhaC*-R	AGCGGCAGGCTAATGTGTAA	
*npr*-F	TCACATCCGTCCAGCATACG	114
*npr*-R	CATGTTGAAAGGACTGCGCC	
*nox2*-F	ATCTTAGACGGTCAGGGCGA	195
*nox2*-R	CGTCGGTAAGGAGGTTGCAG	
*Ldh*-F	GCAGAAGCCAAGGGCATTTC	199
*Ldh*-R	AGCCGTATTCGCCACTCATT	
*Dld*-F	TTTCTCGTGGTCCGTTGGTT	194
*Dld*-R	GGTGTGAGGCGTTACCAAGA	

### 2.8 Data analysis

Experimental data are presented as mean ± SD of triplicate measurements. Data analysis was performed using SPSS 23.0 (SPSS Inc., USA). Statistical significance differences (*p* < 0.05) between treatments were determined by one-way analysis of variance (ANOVA). All the figures were generated using the Origin Pro software (Origin-Lab, version 2021, USA).

## 3 Results and discussion

### 3.1 Morphology of *Limosilactobacillus fermentum* A51

**Figure 1:**
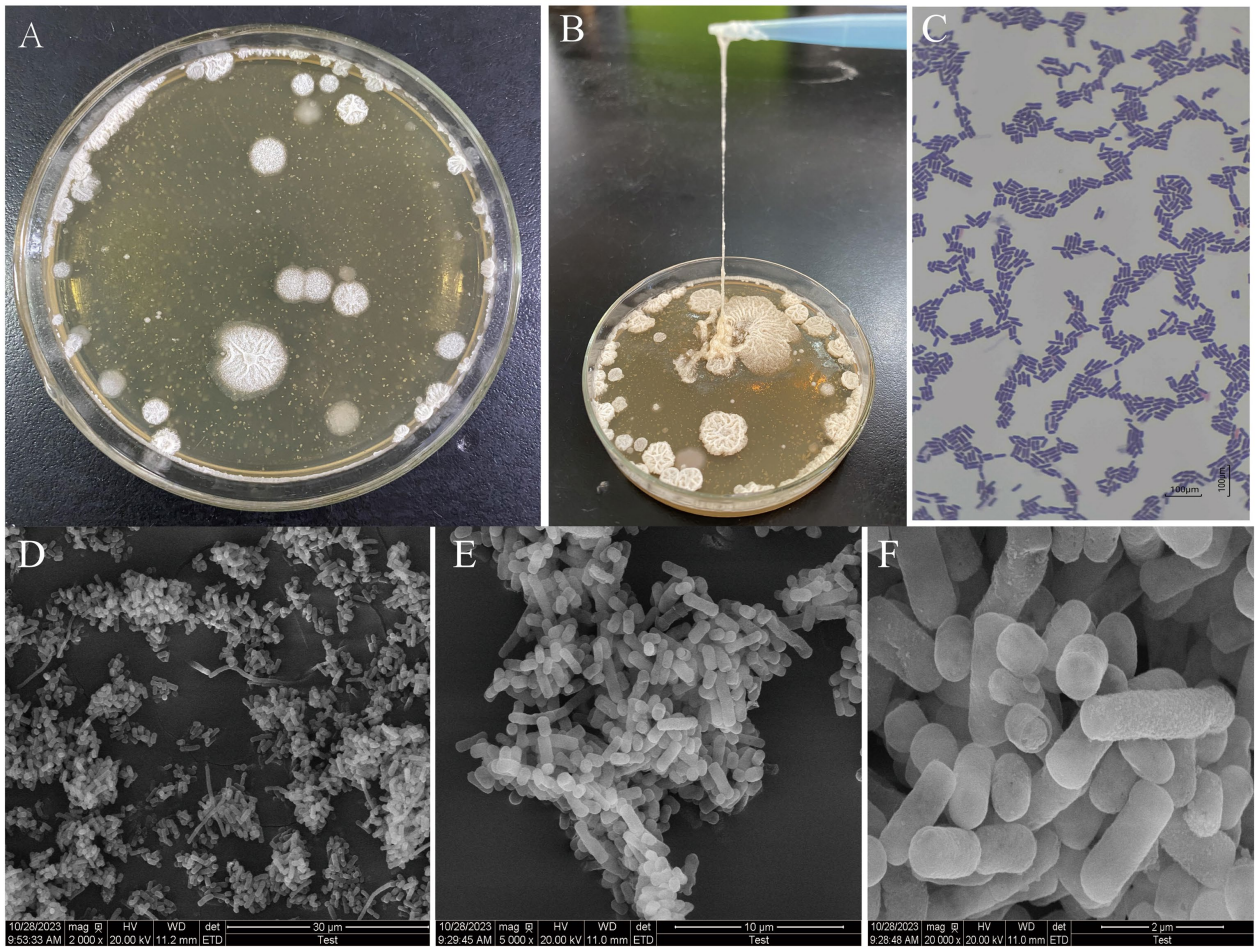
Morphology of *L. fermentum* A51: **(A)** Colony morphology observed on MRS solid plates. **(B)** Colony thread-drawing observed on MRS solid plates. **(C)** Gram-staining; Scanning electron microscope image. **(D)** 2,000 × magnification. **(E)** 5,000 × magnification. **(F)** 20,000 × magnification.

The colony morphology of high EPS-producing LAB grown on MRS solid media is known to be mainly filamentous, mucilaginous, and annular (Xu, 2019). As can be seen in Figures 1A,B, the colony of *L. fermentum* A51 was rounded and greyish-white with neat, elevated, mucilaginous annular morphologies, and had better drawing properties, which was similar to the colony morphology of EPS-producing LAB reported by Ruas-Madiedo and de Los Reyes-Gavilán (2005) and Trabelsi et al. (2015). As shown in Figure 1C, *L. fermentum* A51 could be observed as short purple rods, which is an indication that the strain is Gram-positive bacteria. *L. fermentum* A51 was observed as a slender rod structure, typical morphology of the species under SEM. Interestingly, the colonies distribution was dense, which can be attributed to the secreted EPS of *L. fermentum,* promoting the adhesion of the colonies into clusters (Figures 1D–F; Zhang et al., 2023).

### 3.2 Genome features of *Limosilactobacillus fermentum* A51

#### 3.2.1 General genome features

**Figure 2:**
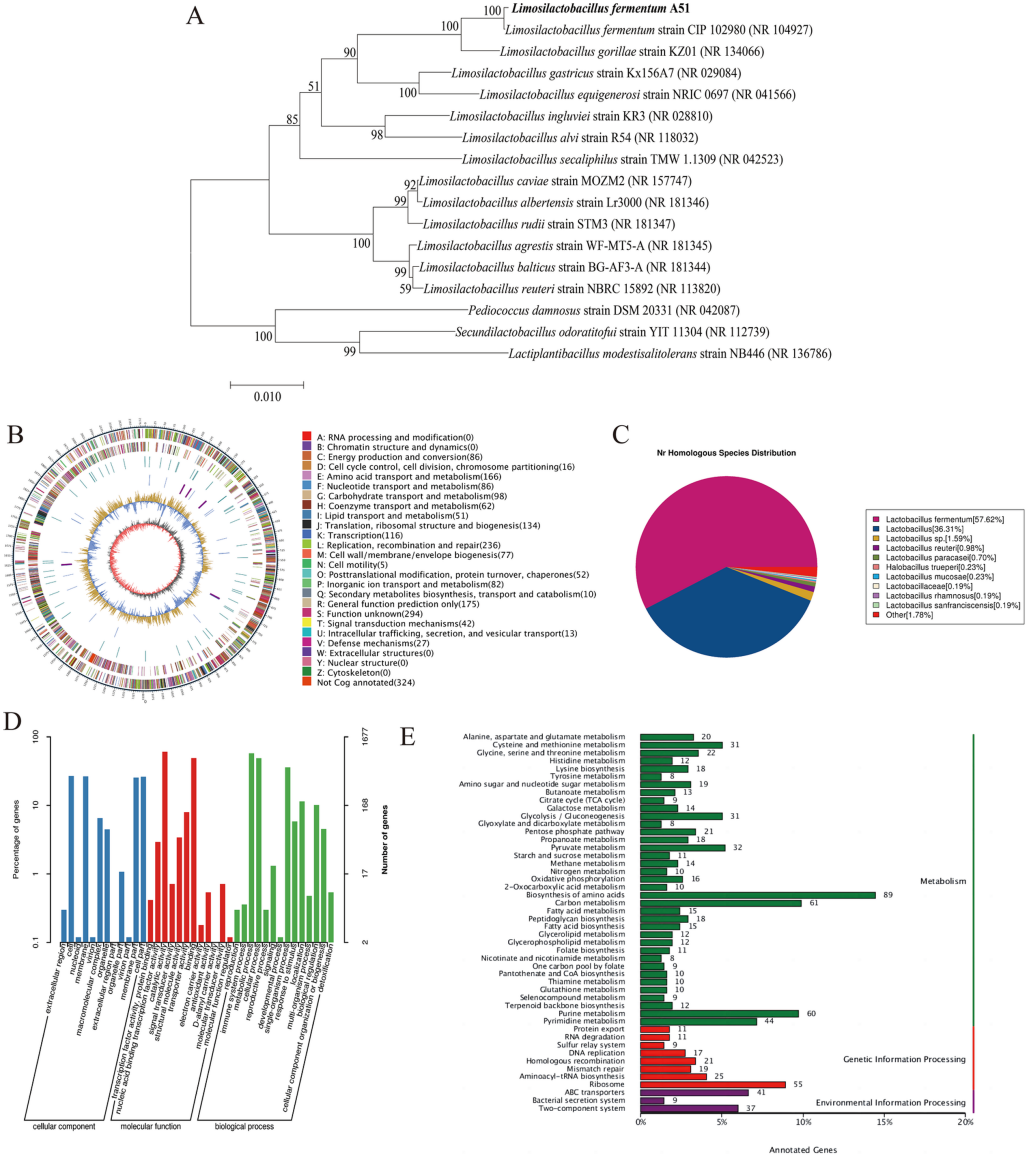
**(A)** Phylogenetic tree was carried out using 1,000 replicates and the number on the nodes represents the support proportion of each branch. **(B)** Circular genome maps. **(C)** NR database-annotated statistics chart. **(D)** GO annotation classification statistics chart, and **(E)** KEGG database annotation of *L. fermentum* A51.

The phylogenetic tree based on 16S rRNA sequences is shown in Figure 2A. The phylogenetic analysis indicated that A51 is closely related to the strain *L. fermentum* CIP 102980. Consequently, A51 was identified as *L. fermentum* and named *Limosilactobacillus fermentum* A51.

In order to obtain a comprehensive understanding of the genome features of *L. fermentum* A51, the whole genome mapping of *L. fermentum* A51 was conducted using PacBio triple sequencing technology, and the sequencing results are shown in Figure 2B and Table 2. The complete genome of *L. fermentum* A51 consisted of a circular chromosome of 2,188,538 bp and had an average gene length of 874 bp and an average GC content of 51.28%. A total of 2,152 genes were predicted by PGAAP and found to include 2,079 protein-coding sequences, 58 rRNAs, 15 tRNAs, and 5 5sRNAs genes. Three incomplete pre-phage sequences with lengths of 85,965 bp, 26,620 bp, and 46,719 bp were predicted in *L. fermentum* A51. The complete genome of *L. fermentum* A51 has been deposited in the NCBI GenBank database (Accession No: CP132542). In addition, four plausible CRISPR regions in *L. fermentum* A51 were predicted. In addition, the NR database annotation results showed that the top 11 species in the NR database with genes matched genes encoding *L. fermentum* A51 were: *Lactobacillus fermentum*, *Lactobacillus* (57.62%), *Lactobacillus* sp. (35.67%), *Lactobacillus reuteri* (1.55%), *Lactobacillus paracasei* (1.01%), *Lactobacillus mucosae* (0.69%), *Halobacillus trueperi* (0.27%), *Lactobacillus rhamnosus* (0.23%), *Lactobacillus sanfranciscensis* (0.18%), *Lactobacillaceae* (0.18%), and other (1.74%) (Figure 2C). Therefore, the strain A51 was identified as *Limosilactobacillus fermentum*, which is consistent with the results of the phylogenetic tree analysis.

**Table 2:** General genome profiles of the *L. fermentum* A51.

Item	Complete genome
Geneset number	2,152
Total length (bp)	2,188,538
Average (bp)	874
Max length (bp)	11,049
Min length (bp)	90
G + C/%	51.28
tRNA NO.	15
rRNA NO.	58
5S rRNA NO.	5
16S rRNA NO.	5
23S rRNA NO.	5
CRISPR Number	4
NR Annotation	2,140
Swissprot_Annotation	1,276
Pfam Annotation	1,811
eggNOG Annotation	1,828
GO Annotation	1,677
KEGG Annotation	1,163

#### 3.2.2 Annotation of functional genes of *Limosilactobacillus fermentum* A51

To further understand the gene function of *L. fermentum* A51, we annotated the identified genes using the GO and KEGG databases. In total, 1,828 genes were annotated by the protein database eggNOG, 1,677 genes were annotated by the GO database, and 1,163 functional genes were annotated by the KEGG database (Table 2). The GO database has an inverted root structure for biological functions, with the three top-level functional nodes being: (1) cellular component (CC), (2) molecular function (MF), and (3) biological process (BP). Among the whole genome sequence of *L. fermentum* A51, 1,677 genes were annotated by the GO database (Figure 2D). These included 1,975 genes related to cellular component CC (427 membrane component, 440 cellular component, 109 macromolecular complex, 75 organelle, and 18 organelle component, etc.), 2,127 genes related to molecular function MF (1,016 catalytic activity, 825 binding, 134 transporter activity, 57 structural molecular activity, 12 signal transducer activity, 9 antioxidant activity, etc.), and 2,976 genes related to biological processes BP (965 metabolism, 816 cellular processes, 602 single organism processes, 170 biomodulation, 98 stress response, 22 signaling, etc.). Although these genes are involved in every part of cellular metabolism, they have different intensities, indicating their involvement of different processes in the cell is different.

There were 1,163 genes in the whole genome sequence of *L. fermentum* A51 annotated by KEGG, of which metabolism-related genes were the most numerous genes, including 60 for purine metabolism, 44 for pyrimidine metabolism, 32 for pyruvate metabolism, amino acid metabolism (pyruvate metabolism 31, glycine, serine and threonine metabolism 22, alanine, aspartate and glutamate metabolism 20, histidine metabolism 12), and gluconeogenesis (glycolysis/gluconeogenesis 31, pentose phosphate pathway 21, galactose metabolism 14 and phosphotransferase system (PTS) 7). These numbers show that the bacterium has a strong protein and sugar catabolism and metabolism function (Liu et al., 2022). In addition, biosynthesis-related genes were also annotated by KEGG: 89 genes for amino acid biosynthesis, 18 for lysine biosynthesis, 18 for peptidoglycan biosynthesis, 15 for fatty acid biosynthesis, 12 for arginine biosynthesis and 11 for folate biosynthesis (Figure 2E).

### 3.3 Safety assessment of *Limosilactobacillus fermentum* A51

#### 3.3.1 Antibiotic sensitivity

As potential probiotics, LAB should not exhibit transferable antibiotic resistance. In the current study, the susceptibility of *L. fermentum* A51 to ten common antibiotics was determined (Table 3). The results show that *L. fermentum* A51 is sensitive to erythromycin, chloromycetin, tetracycline, ampicillin, and gentamicin, intermediate to clindamycin and ofloxacin, resistant against ciprofloxacin, kanamycin, and vancomycin. Lactobacilli are resistant to vancomycin because their peptidoglycan contains D-Ala-D-lactic acid rather than D-Ala-D-Ala dipeptide (Oh et al., 2022). Notably, some studies have confirmed that many of the resistance attributes are inherent and cannot be transferred (Salminen et al., 1998). Thus, *L. fermentum* A51 is considered safe.

**Table 3:** Judgment of antibiotic susceptibility level of *L. fermentum* A51.

Name	Dosage/μg	Standard			Bacteriostatic zone diameter/mm	Degree of sensitivity
		S	I	R		
Erythromycin	15	≥23	14–22	≤13	26.19 ± 0.62	S
Chloromycetin	30	≥18	13–17	≤12	24.38 ± 1.27	S
Tetracycline	30	≥19	15–18	≤14	20.37 ± 1.03	S
Ciprofloxacin	5	≥21	16–20	≤15	11.02 ± 0.92	R
Clindamycin	20	≥21	15–20	≤14	19.37 ± 0.84	I
Ampicillin	10	≥17	14–16	≤13	24.73 ± 1.26	S
Gentamicin	10	≥15	13–14	≤12	16.83 ± 1.63	S
Kanamycin	30	≥18	14–17	≤13	10.11 ± 0.65	R
Vancomycin	30	≥17	15–16	≤14	0	R
Ofloxacin	5	≥16	13–15	≤12	13.92 ± 1.26	I

S means sensitive; I means intermediate; R means resistance.

#### 3.3.2 Hemolytic activity

The hemolytic activity of probiotics is one of the most important criteria for their safety assessment. The hemolysis assay showed that a transparent ring was observed around the bacteria colonies of *S. aureus* CICC 10384 (positive control), which indicates *β*-hemolysis (Supplementary Figure S1C). A grass-green haemolytic ring found around the bacteria colonies of *E. coli*, indicating *α*-hemolysis (Supplementary Figure S1D). While, no hemolytic ring found around the bacteria colonies of *L. fermentum* A51 (Supplementary Figure S1A), which was consistent with the control strain *Lactobacillus casei* Zhang (Supplementary Figure S1B). Therefore, we considered the *L. fermentum* A51 to be *γ*-hemolytic, which is consistent with the results of Wang et al. (2023), who reported that *Limosilactobacillus fermentum* MWLf-4 from human milk was γ-hemolytic. This finding suggests that *L. fermentum* A51 is safe for human applications based on its hemolytic activity.

#### 3.3.3 Antibiotic resistance genes of *Limosilactobacillus fermentum* A51

The CARD database search showed that *L. fermentum* A51 has only one potential antibiotic resistance gene (*poxtA*) that is resistant to tetracycline, oxazolidinone, and phenicol. However, the results of the antibiotic resistance test showed that *L. fermentum* A51 is sensitive to tetracycline, indicating that the tetracycline-encoding gene may not be expressed. In addition to the phenotypic results of the antibiotic resistance test, these findings suggest that *L. fermentum* A51 is not resistant to common antibiotics. This may be related to the origin of the strain. *L. fermentum* A51 is derived from fermented yak milk, which is made from fresh yak milk, sterilized, fermented, and post-cooked without the addition of preservatives during production.

#### 3.3.4 Virulence factor genes of *Limosilactobacillus fermentum* A51

The virulence factor gene in *L. fermentum* A51 was annotated through the VFDB database. A total of 290 potential virulence factor genes were annotated in *L. fermentum* A5. However, most of the putative virulence factor genes exhibited less than 50% similarity to those in the VFDB database, and only five genes were annotated with similarity greater than 60% (but all≤75%) (Supplementary data 1). In addition, no genes encoding hemolysins (*cylA*, *cylB*, *cylM*, *Hbl*, *Nhe*, and *cereulide-Ces*) were identified in the *L. fermentum* A51 genome, which is consistent with the absence of hemolytic activity. Based on the annotation of the KEGG and COG functional databases, most of these putative virulence genes were annotated as genes involved in carbohydrate transport, ABC transport systems, two-component systems, bacterial adhesion, stress response, and quorum sensing systems. ABC transport system plays a key role in normal substance transportation and release of exogenous toxic compounds and waste metabolites *in vivo* (Zhu et al., 2019). The two-component system is involved in the regulation of various physiological and biochemical functions of lactic acid bacteria, which is an important regulatory system for their metabolic activities. In addition, most of these putative virulence genes were associated with carbohydrate transport, indicating that *L. fermentum* A51 has a strong sugar metabolism ability (Choi et al., 2008). The annotated adhesion genes allow *L. fermentum* A51 to better adhere to and colonize the host cells (Altermann et al., 2005; Senan et al., 2015). Meanwhile, some virulence factor genes are associated with probiotic functions in LAB. For example, the *luxS* gene is associated with the LuxS/AI-2 type quorum sensing system in LAB, which regulates tolerance and adhesion of LAB (Meng et al., 2022). The gene *bsh* is positively associated with bile salt tolerance in LAB (Choi and Chang, 2015). In pathogenic bacteria, genes associated with biofilm formation may be identified as pathogens. However, for LAB, these genes are associated with adhesion and resistance (Meng et al., 2022). Therefore, based on the results of hemolysis and antibiotic resistance tests, as well as the analysis of genes involved in antibiotic resistance, virulence factor, and hemolytic activity assay, we can postulate that *L. fermentum* A51 is safe.

### 3.4 Probiotic gene features of *Limosilactobacillus fermentum* A51

#### 3.4.1 Genes related to stress response of *Limosilactobacillus fermentum* A51

A prerequisite that allows probiotics to exhibit its phenotypic characteristics is their ability to survive the passage through the human gastrointestinal tract. Therefore, LAB need to adapt to their acidic fermentation environment and also tolerate the acidic conditions and bile salt of the gastrointestinal tract. The survival rate of *L. fermentum* A51 in bile salt and *in-vitro* simulated gastrointestinal digestion is shown in Tables 4, 5. After treated in 0.3% bile salt for 4 h, the survival rate of *L. fermentum* A51 was 39.06%, which was lower than that of *L. casei* Zhang (43. 47%) but higher than that of *L. plantarum* ZFM4 (35.5%) (Yan et al., 2023), suggesting that *L. fermentum* A51 has better bile salt tolerance (Table 4). After digestion with simulated gastric juice for 2 h, the viable counts of *L. fermentum* A51 and *L. casei Zhang* (control) were 70.87% (viable counts was 8.70 log CFU/mL) and 76.67% (viable counts was 9.43 log CFU/mL), respectively, which were not significantly different (*p* < 0.05; Table 5). After being exposed to intestinal fluid for 2 h, the survival rates of *L. fermentum* A51 and *L. casei* Zhang decreased to 64.56% (viable counts was 8.49 log CFU/mL) and 70.34% (viable counts was 9.32 log CFU/mL) respectively. After gastric juice and intestinal fluid treatment, the viable counts of strains remained above 8 log CFU/mL, indicating that both strains were well tolerated. However, the tolerability to the gastrointestinal environment of *L. casei* Zhang is slightly better than that of *L. fermentum* A51. Previous studies have shown that EPS-producing LAB are more resistant and tolerant to bile salts than free LAB (Gu et al., 2020). *L. casei* Zhang is a highly EPS-producing strain (546.3 ± 31.5 mg/L; Ba et al., 2021). The gastrointestinal digestive tolerance of *L. fermentum* A51 and *L. casei* Zhang is related to their EPS production capacity. EPS produced by these two strains wrapped around the strains, forming a protective layer that enhances their environmental tolerance. In addition, bile salt hydrolase (Bsh) and choloylglycine hydrolase family protein (Ntn) produced in some LAB are important for the survival of the bacteria in the intestinal tract (Choi and Chang, 2015). Cyclopropane-fatty-acyl-phospholipid synthase (CFA), cystathionine-*β*-lyase, and cystathionine-*γ*-synthase protect LAB from adverse conditions such as acidity (Ma et al., 2019; Charnchai et al., 2017). Na^+^/H^+^-antiporter acts as a regulator of intracellular pH, enabling the bacteria to survive in an acidic environment (Iyer et al., 2002). Genes encoding bile salt hydrolase (*Bsh*), Na^+^/H^+^-antiporter (*nhaC*), choloylglycine hydrolase family protein (*Ntn*), cyclopropane-fatty-acyl-phospholipid synthase (*cfa*), and ATPase (*sufC*) were found in the *L. fermentum* A51 genome. The presence of these genes suggests that *L. fermentum* A51 can tolerate bile salts and acids (Table 6).

**Table 4:** Tolerance of *L. fermentum* A51 to bile salt.

Strains	Strain survival rate %			
	Bile salt concentration 0.1%	Bile salt concentration 0.2%	Bile salt concentration 0.3%	Bile salt concentration 0.4%
*L. fermentum* A51	56.28 ± 2.37^b^	47.83 ± 1.92^b^	39.06 ± 2.33^b^	27.47 ± 3.49^b^
*L. casei Zhang*	61.03 ± 3.92^a^	56.63 ± 2.44^a^	43.47 ± 3.06^a^	29.34 ± 2.11^a^

Different superscript letters differ significantly between the columns (*p* < 0.05).

**Table 5:** Tolerance of *L. fermentum* A51 to gastrointestinal fluid.

Strains	0 h live bacteria (log CFU/mL)	Viable bacteria after 2 h gastric Juice treatment (logCFU/mL)	Survival rate (%)	Viable bacteria after 2 h intestinal fluid treatment (log CFU/mL)	Survival rate (%)
*L. fermentum* A51	8.85 ± 0.06^b^	8.70 ± 0.07^b^	70.87 ± 2.95^b^	8.49 ± 0.07^b^	64.56 ± 3.54^b^
*L. casei Zhang*	9.54 ± 0.07^a^	9.43 ± 0.06^a^	76.67 ± 2.57^a^	9.32 ± 0.13^a^	70.34 ± 0.97^a^

Different superscript letters differ significantly between the columns (*p* < 0.05).

**Table 6:** Representative genes related to probiotic characteristics in *L. fermentum* A51.

Gene ID	Gene	Function annotation
Genes related to stress response		
GE000498	*atp*C	ATP synthase epsilon chain
GE001382	*-*	Fibronectin/fibrinogen-binding protein
GE000375	*groEL*	Chaperonin GroEL
GE000374	*groES*	Co-chaperone GroES
GE000679	*comGC*	Competence protein ComGC
GE000774	*tuf*	Elongation factor Tu
GE000226	*srtA*	Sortase A
GE000031	*Ntn*	Choloylglycine hydrolase family protein
GE000117	*cfa*	Cyclopropane-fatty-acyl-phospholipid synthase
GE000031, GE001146	*bsh*	Bile salt hydrolase, choloylglycine Hydrolase
GE001061	*sufC*	ATPase
GE001682	*nhaC*	Na+/H+-antiporter
GE000938	*dnaJ*	Molecular chaperone DnaJ
GE000937	*dnaK*	Molecular chaperone DnaK
GE001594	*HSP20*	Hsp20/alpha crystallin family protein
GE000935	*hrcA*	Heat-inducible transcriptional repressor HrcA
GE000036	*clpL*	ATP-dependent Clp protease, ClpL
GE000936	*grpE*	Nucleotide exchange factor GrpE
GE001726	*clpP*	ATP-dependent Clp protease proteolytic subunit
GE000508	*-*	Universal stress protein
GE000791	*-*	Universal stress protein
GE001379	*-*	Universal stress protein
GE001573	*-*	Universal stress protein
GE001822	*-*	Universal stress protein
GE002021	*-*	Universal stress protein
GE002069	*-*	Universal stress protein
GE001726	*clpP*	ATP-dependent Clp protease proteolytic subunit
Genes related to antioxidant activity		
GE000975	*msrA*	Peptide methionine sulfoxide reductase MsrA
GE001108	*msrB*	Peptide methionine sulfoxide reductase MsrB
GE001277	*nrfA*	FMN reductase (NADPH)
GE001685	*npr*	NADH peroxidase
GE000526	*tps*	Thiol peroxidase, atypical 2-Cys peroxiredoxin
GE001901	*trsX*	Thioredoxin 1
GE001235	*nox2*	NADH flavin oxidoreductase
GE001900	*gor*	glutathione reductase
GE001216	*gshAB*	Glutathione biosynthesis bifunctional protein GshAB
GE000396	*trxB*	Thioredoxin-disulfide reductase
GE000329	*nrdH*	Glutaredoxin-like protein NrdH
GE001999	*qorB*	NAD(P)H dehydrogenase (quinone)
GE002135	*mntH*	Manganese transport protein
GE001318	*fitE*	Peptide ABC transporter substrate-binding protein
Genes related to antibacterial activity		
GE000805, GE000996	*ldh*	L-lactate dehydrogenase (phenyllactic acid synthesis)
GE000033, GE000788, GE001329, GE002077	*dld*	D-lactate dehydrogenase (phenyllactic acid synthesis)
GE000556	*-*	Colicin V production protein (bacteriocin related genes)
Genes related to EPS synthesis		
GE001643	*glf*	UDP-galactopyranose mutase
GE000103	*epsG*	Controlled repeat unit synthesis
GE000104	*gtf*	Glycosyl transferase
GE001628	*rfbB*	dTDP-glucose 4,6-dehydratase
GE001642	*Wzx*	Flippase
GE001644, GE000096	*Wzz*	Membrane/envelope biogenesis
GE001645	*Wzy*	Polymerase
GE000919	*malY*	Maltose/moltooligosaccharide transporter
GE001609	*-*	Glycosyltransferase 2 family protein
Genes related to signal molecule AI-2 synthesis		
GE001633	*Metk*	S-adenosylmethionine synthetase
GE000705	*DNMT1/dcm*	S-adenosylmethionine-dependent methyltransferase
GE000728	*pfs*	S-adenosylhomocysteine nucleosidase
GE001884	*luxS*	S-ribosylhomocysteine lyase
GE001072	*mmuM*	Homocysteine S-methyltransferase

#### 3.4.2 Adhesion capacity-related genes

**Figure 3:**
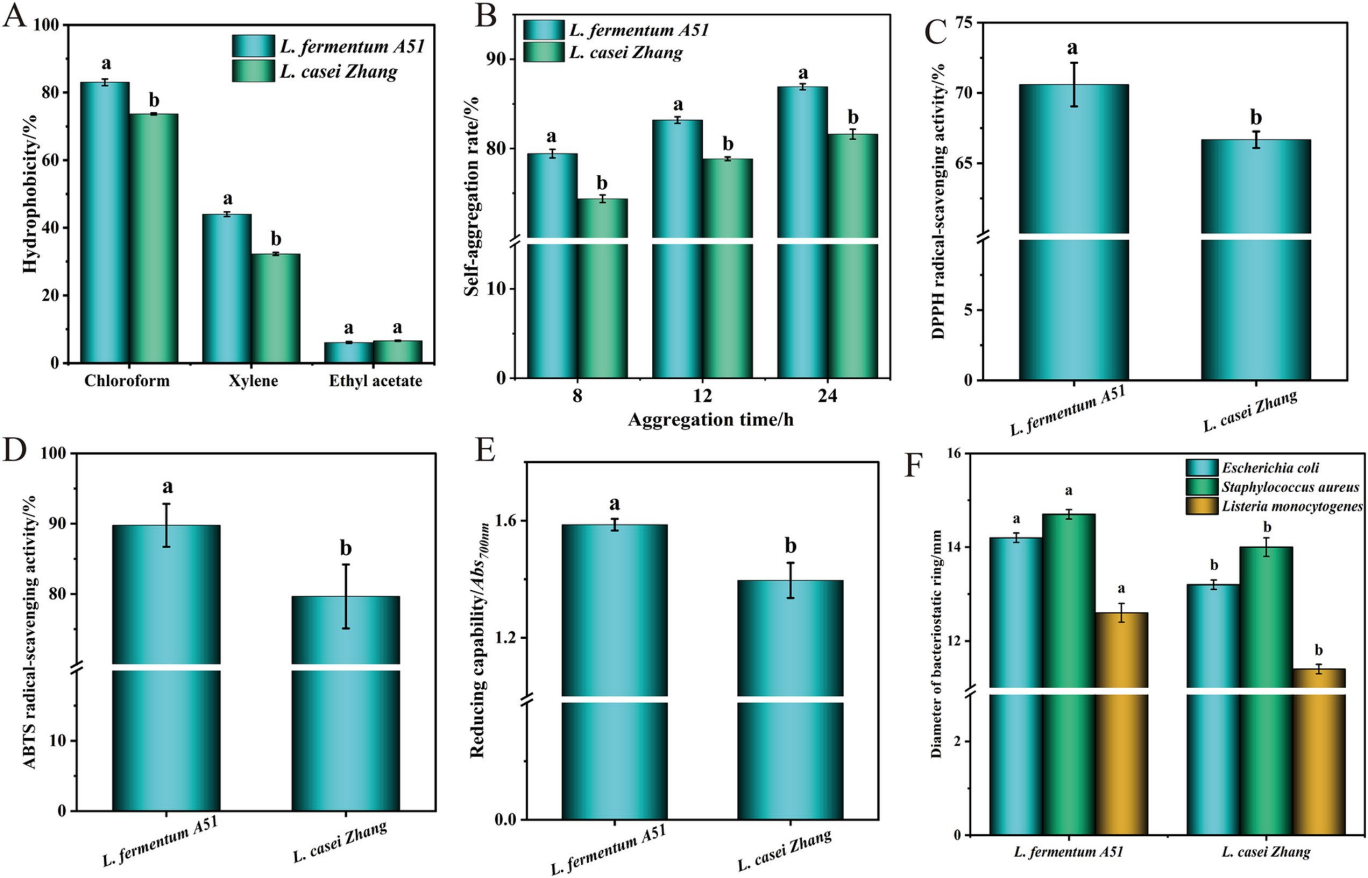
Probiotic properties of *L. fermentum* A51. **(A)** Hydrophobic capability. **(B)** Self aggregation capability. **(C)** DPPH free radical scavenging activity. **(D)** ABTS free radical scavenging activity. **(E)** Reducing capability. **(F)** Inhibitory activity against *E. coli* and *S. aureus.* Different lower case letters represent significant differences with *p* < 0.05 between *L. fermentum* A51 and *L. casei* Zhang.

Hydrophobicity and autoaggregation capability are the evaluation indexes of non-specific adhesion of LAB to the intestine (Peng et al., 2023). The hydrophobicity and autoaggregation capability of *L. fermentum* A51 was measured (Figures 3A,B). As shown in Figure 3A, the hydrophobicity of *L. fermentum* A51 in chloroform, xylene, and ethyl acetate were all significantly higher than that of *L. casei* Zhang (*p* < 0.05). In particular, *L. fermentum* A51 had the highest hydrophobicity of 83.03 ± 0.08% in chloroform. The autoaggregation rate of the strains all showed an increasing trend with the increase in adhesion time (Figure 3B). However, the autoaggregation capability of *L. fermentum* A51 remained higher than that of *L. casei* Zhang. At 24 h, the autoaggregation capability of *L. fermentum* A51 was 86.92 ± 0.34% (>50%), indicating it had good autoaggregation capability (Montoro et al., 2016). Whole-genome sequencing analysis showed that *L. fermentum* A51 contained adhesion-related genes, including genes encoding fibronectin/fibrinogen-binding protein, sortase A (*srtA*), competence protein ComGC (*ComGC*), chaperonin GroEL (*GroEL*) and elongation factor Tu (*tuf*), which allows *L. fermentum* A51 to better adhere to and colonize in the host cells (Altermann et al., 2005; Senan et al., 2015; Table 6).

#### 3.4.3 Antioxidant activity-related genes

Many studies have shown that LAB has antioxidant capacity. In the current study, the *in vitro* antioxidant activity of *L. fermentum* A51 was measured, and the results are shown in Figures 3C–E. As shown in Figure 3C, the CFS of *L. fermentum* A51 showed that it had a DPPH radical scavenging activity of 70.60%, which is significantly higher (*p* < 0.05) than that of the control (66.67% for *L. casei Zhang* and 28.02% for *Lactiplantibacillus plantarumisolated* D444 from Chinese traditional fermented foods) (Zhang et al., 2010; Sun et al., 2022). In addition, the ABTS radical scavenging activity and reducing capability of *L. fermentum* A51 were significantly stronger than that of the control (*p* < 0.05) (Figures 3D–E), indicating that *L. fermentum* A51 has a strong antioxidant capacity. The antioxidant activity of *L. fermentum* A51 may be due to its ability to produce superoxide dismutase (SOD), reduced glutathione (GSH), ferulic acid esterase (FAE), ferulic acid (FA), NADH oxidase and NADH peroxidase (Cheng et al., 2020). Whole genome mapping analysis showed that *L. fermentum* A51 carried genes encoding thioredoxin reductase (*NADPH*), glutathione reductase, peptide methionine sulfoxide reductase msrA/msrB (*msrA*, *msrB*), thiol peroxidase (*tps*) and thioredoxin 1 (*trsX*) (Table 6), implying that *L. fermentum* A51 possesses antioxidant activity (Papadimitriou et al., 2016). NADH oxidase is directly involved in detoxifying hydrogen peroxide and ROS. The antioxidant enzyme glutathione reductase is a crucial antioxidant enzyme responsible for the maintenance of glutathione, one of the main antioxidant metabolites (Liang et al., 2020). The expression of thioredoxin genes can increase the activity of antioxidant enzymes, such as superoxide dismutase (SOD) and catalase (CAT). In addition, under certain conditions, thioredoxin can promote the conversion of oxidized glutathione to reduced glutathione (Shi et al., 2010). For non-enzymatic antioxidants, probiotics secreting EPS and antioxidant peptides (e.g., glutathione) can reduce oxidative damage and thus can prevent aging and various chronic diseases (Zhang et al., 2022; Xue et al., 2023). All the above findings indicate that *L. fermentum* A51 is a potential probiotic with antioxidant ability.

#### 3.4.4 Antibacterial activity-related genes

The antibacterial activity of *L. fermentum* A51 against *E. coli*, *S. aureus* and *L. monocytogenes* was evaluated, and the results are shown in Figure 3F. The diameters of the inhibitory zones of *L. fermentum* A51 against *E. coli*, *S. aureus* and *L. monocytogene* were 14.2 ± 0.1 mm, 14.7 ± 0.1 mm and 12.6 ± 0.2 mm, respectively, which were higher (*p* < 0.05) than those of control (*L. casei Zhang*, 13.2 ± 0.1 mm, 14.0 ± 0.2 mm and 11.4 ± 0.1 mm). The antibacterial capacity of *L. fermentum* A51 is possibly related to the protein and non-protein antibacterial components, such as bacteriocins, organic acids, hydrogen peroxide and EPS, generated by the strain. Organic acid antibacterial substances, including lactic acid, phenyl lactic acid, citric acid, and acetic acid, are metabolized by LAB during fermentation. Organic acids inhibit the growth of pathogenic bacteria by increasing their outer membrane permeability, altering intracellular osmotic pressure, and inhibiting DNA synthesis. Preliminary experiments demonstrated that the antimicrobial substances in *L. fermentum* A51 were not bacteriocins, hydrogen peroxide, and EPS, but organic acids. Moreover, *L. fermentum* A51 contained genes involved in phenyl lactate synthesis (two *ldh* genes and four *dld* genes) (Table 6). Therefore, it can be assumed that the inhibitory effect of *L. fermentum* A51 against *E. coli* and *S. aureus* is also due to organic acids. Overall, these findings indicate that *ldh* and *dld* genes identified in the genome of *L. fermentum* A51 may help in its antimicrobial activity (Zhou et al., 2020).

#### 3.4.5 Genes involved EPS synthesis

**Figure 4:**
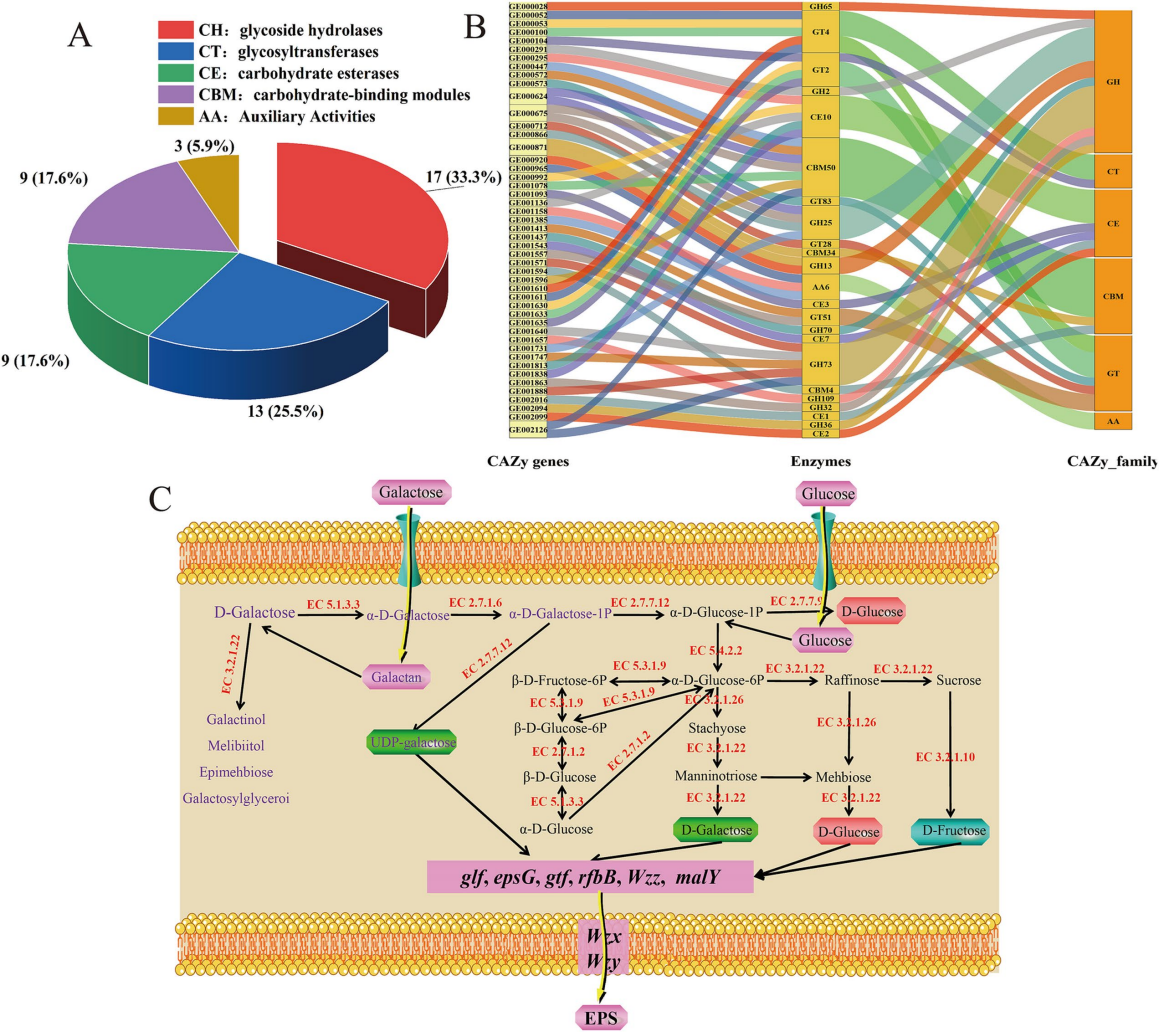
Prediction of genes encoding carbohydrate-active enzymes prediction. **(A)** Carbohydrate-active enzymes database (CAZy)-annotated statistics chart. **(B)** Classification of predicted CAZy from *L. fermentum* A51. **(C)** Carbohydrate metabolic systems in *L. fermentum* A51 genome. “EC + number” indicates the annotation of enzymes in *L. fermentum* A51. EPS, exopolysaccharides.

Fifty-one carbohydrate active enzyme genes in the whole genome sequence of *L. fermentum* A51 were annotated by CAZy, including auxiliary oxidoreductases (AAs, 3 genes), carbohydrate esterases (CEs, 9 genes), functional domains of carbohydrate-binding modules (CBMs, 9 genes), glycoside hydrolases (GHs, 17 genes) and glycosyltransferases (GTs, 13 genes) (Figures 4A,B). However, the annotation did not indicate polysaccharide lyase (PLs) family genes in the *L. fermentum* A51 genome. GHs have the potential to hydrolyze complex carbohydrates and are considered to be key enzymes responsible for carbohydrate metabolism. The 17 GH genes in *L. fermentum* A51 were predicted to belong to nine different families, according to the CAZy database, which suggests that *L. fermentum* A51 can utilize a wide range of carbohydrates. Alpha-galactosidase [EC 3.2.1.22] and beta-galactosidase [EC 3.2.1.23], members of the GH2 and GH36 family, are responsible for the metabolism of D-galactose and lactose (Su et al., 2010; Graebin et al., 2016). In this study, a variety of genes encoding GTs were found in *L. fermentum* A51, with GT2 and GT4 being the most abundant genes. GT2 and GT4 enzymes are associated with the synthesis of disaccharides such as sucrose, as well as the synthesis of lipopolysaccharides, cellulose, and chitosan. The high content of GT2 and GT4 (galactosyltransferase, glucosyltransferase, rhamnosyltransferase, and N-acetylglucosaminyltransferase [EC 2.4.1]) indicates that *L. fermentum* A51 has a high capacity in sugar and EPS synthesis. In summary, alpha/beta-glucosidases hydrolyze the glucosidic bonds to produce monosaccharides, providing precursor materials for EPS biosynthesis, whereas glycosyltransferases are responsible for transferring EPS from the intracellular space to the extracellular matrix. The KEGG annotation also identified *L. fermentum* A51 as rich in carbohydrate transport-and metabolism-related genes, including glycolysis/gluconeogenesis genes (31), phosphotransferase system genes (21) and galactose metabolism genes (14) (Supplementary Table S1). In particular, *L. fermentum* A51 possesses a complete galactose and glucose metabolic pathway. It can be implied that *L. fermentum* A51 can synthesize EPS using galactose and glucose and thus could potentially be used in value-added fermented dairy products (Figure 4C).

Whole-genome sequencing analysis showed that *L. fermentum* A51 contained EPS synthesis-regulating genes, including *glf* (GE001643), *epsG* (GE000103), *Wzx* (GE001642), *Wzz* (GE001644), *Wzy* (GE001645), *pgk* (GE000425) and *glf* (GE001643), suggesting that EPS synthesis by *L. fermentum* A51 relies on the Wzx/wzy-dependent pathway (Wu et al., 2022; Figure 4C; Table 6). The steps for the synthesis of EPS by *L. fermentum* A51 were as follows: phosphotransferases transfer extracellular glucose and galactose into the cell for phosphorylation. The *pgk* and *glf* genes are involved in the production of sugar nucleotides. The *epsG* genes encode glycosyltransferases, which are responsible for the translocation of monosaccharides to lipid carriers and the synthesis of repeating units. In the polymerization of repeats, the chain length-determining protein, *Wzz*, controls the length of the polysaccharide chain by controlling the number of repeats that are added. The repeating units are transferred by flippase to the periplasmic space or outer membranes. The repeating units are polymerized into long chains by Wzy polymerase. Eventually, the long-chain EPS is released into the extracellular space by Wzx flippase. Genotypic analysis showed that *L. fermentum* A51 was rich in carbohydrate transport and metabolism-related genes, as well as key EPS synthesis regulating genes. These genes allow *L. fermentum* A51 to synthesize EPS.

**Figure 5:**
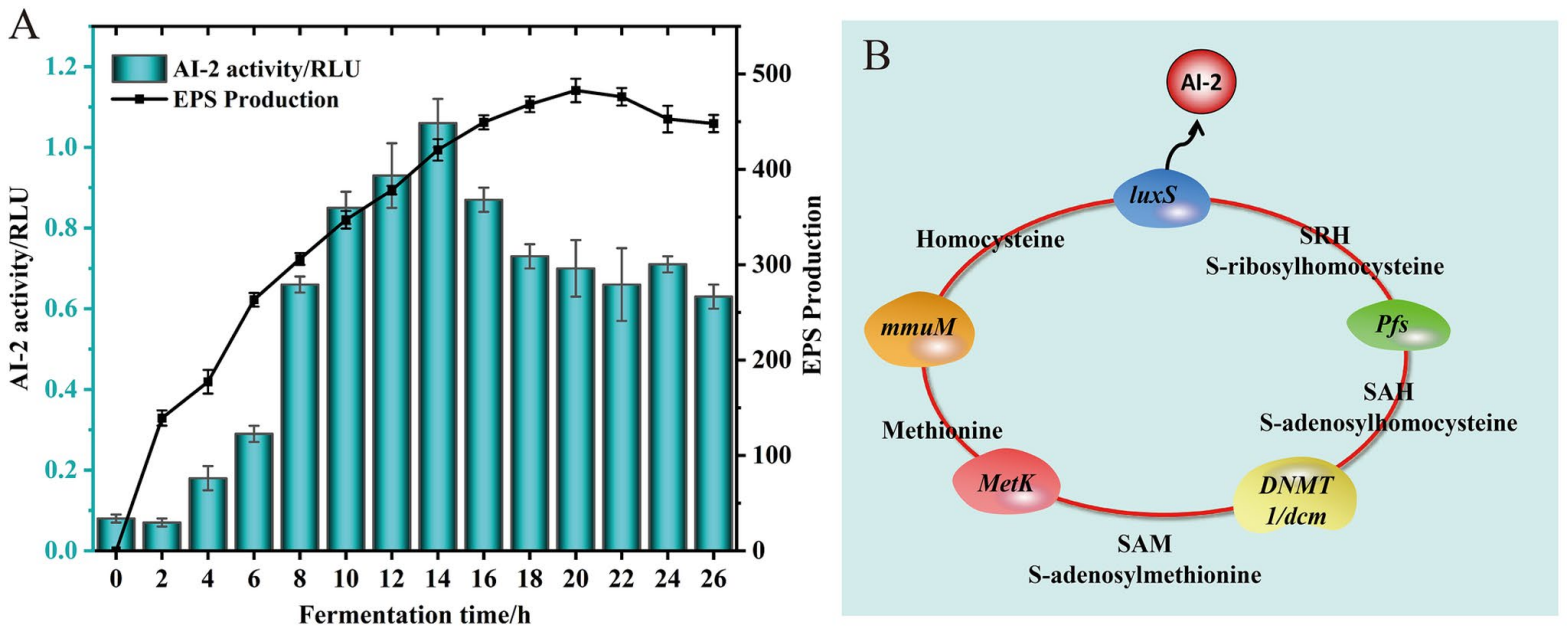
EPS production and AI-2 activity of *L. fermentum* A51 at different culture time **(A)**. Signaling molecule AI-2 synthesis pathway of *L. fermentum* A51 **(B)**.

The EPS synthesis capability of *L. fermentum* A51 in MRS liquid medium was determined, and the results are shown in Figure 5A. The production of EPS increased significantly during the first 14 h and reached a maximum value of 482.728 ± 12.304 mg/L at 20 h, but then decreased as fermentation progressed further (Figure 5A). The decrease of EPS production in late fermentation may be caused by the degradation of glycohydrolases presented in the culture for EPS synthesis. Our results agree with the findings of Li M. et al. (2022), who found that EPS production of *Lactococcus lactis* subsp. *lactis* IMAU11823 in MRS liquid medium increased at the fastest rate during the first 12 h and gradually increased to a maximum value of 201.44 mg/L at 24 h.

#### 3.4.6 LuxS/AI-2 quorum sensing system-related genes

Recently, several studies have reported that the probiotic properties of LAB, which include resistance to harsh environmental conditions, biofilm formation, phenyl lactic acid synthesis, conjugated linoleic acid production, adhesion and colonization, and bacteriocin synthesis are closely related to the regulation of the LuxS/AI-2 quorum sensing (QS) system (Ma et al., 2022; Meng et al., 2022; Chai et al., 2023). The LuxS/AI-2-QS system is a density-dependent regulatory system that relies on a cascade reaction to regulate the expression of target genes. This cascade response is triggered by an extracellular signaling molecule, AI-2, at a certain threshold concentration (Moreno-Gámez et al., 2017). The activity of signaling molecule AI-2 during fermentation of *L. fermentum* A51 was determined (Figure 5A). During the first 12 h of fermentation, the fluorescence intensity of the signal molecule AI-2 produced by *L. fermentum* A51 gradually increased. Starting from 12 h, the signal molecule AI-2 was higher than the positive relative fluorescence intensity, demonstrating that *L. fermentum* A51 could produce the signal molecule AI-2. The activity of the signal molecule AI-2 was highest at 14 h of incubation and then decreased with increasing incubation time. The reduced activity of the signal molecule AI-2 at a later stage may be related to the unstable and easily decomposed character of the signal molecule AI-2 (Chen et al., 2002).

Genomic results showed that *L. fermentum* A51 contained complete genes involved in the synthesis of signaling molecule AI-2, including *luxS* (GE001943), *pfs* (GE000728), *DNMT1/dcm* (GE000705), *MetK* (GE001633), and *mmuM* (GE001072) genes (Figure 5B; Table 6). This finding suggests that *L. fermentum* A5 has the ability to secrete the signaling molecule AI-2 (Duanis-Assaf et al., 2016). The secreted signaling molecule AI-2 can then activate the LuxS/AI-2-QS system. Whole genome sequencing analysis and phenotypic experiments confirmed that *L. fermentum* A51 showed good tolerance to simulated gastrointestinal tract and possessed antioxidant and antimicrobial activities, as well as EPS synthesis capacities. Previous studies have indicated that the LuxS/AI-2-QS system positively regulates the expression of the *nhaC* gene (Na^+^/H^+^ reverse transporter protein) and enhances the survival of *Lactobacillus acidophilus* CICC 6074 in intestinal juice (Li X. et al., 2022). Another study discovered that the LuxS/AI-2-QS system mediates the synthesis of phenyl lactic acid in *L. plantarum* L3 by regulating the expression of key proteins, including S-ribosomal homocysteine lyase (LuxS), aminotransferase (araT) and lactate dehydrogenase (ldh) (Chai et al., 2023). Wang (2019) demonstrated that the LuxS/AI-2-QS system mediates the expression of S-layer proteins of *Lactobacillus acidophilus* CICC6074 and its adhesion to the intestine. In this study, the LuxS/AI-2-QS system was positively associated with the probiotic properties of LAB. However, the regulatory relationship between the LuxS/AI-2-QS system and the probiotic properties of *L. fermentum* A51 needs to be further investigated.

### 3.5 Validation of probiotic genes using RT-qPCR

**Figure 6:**
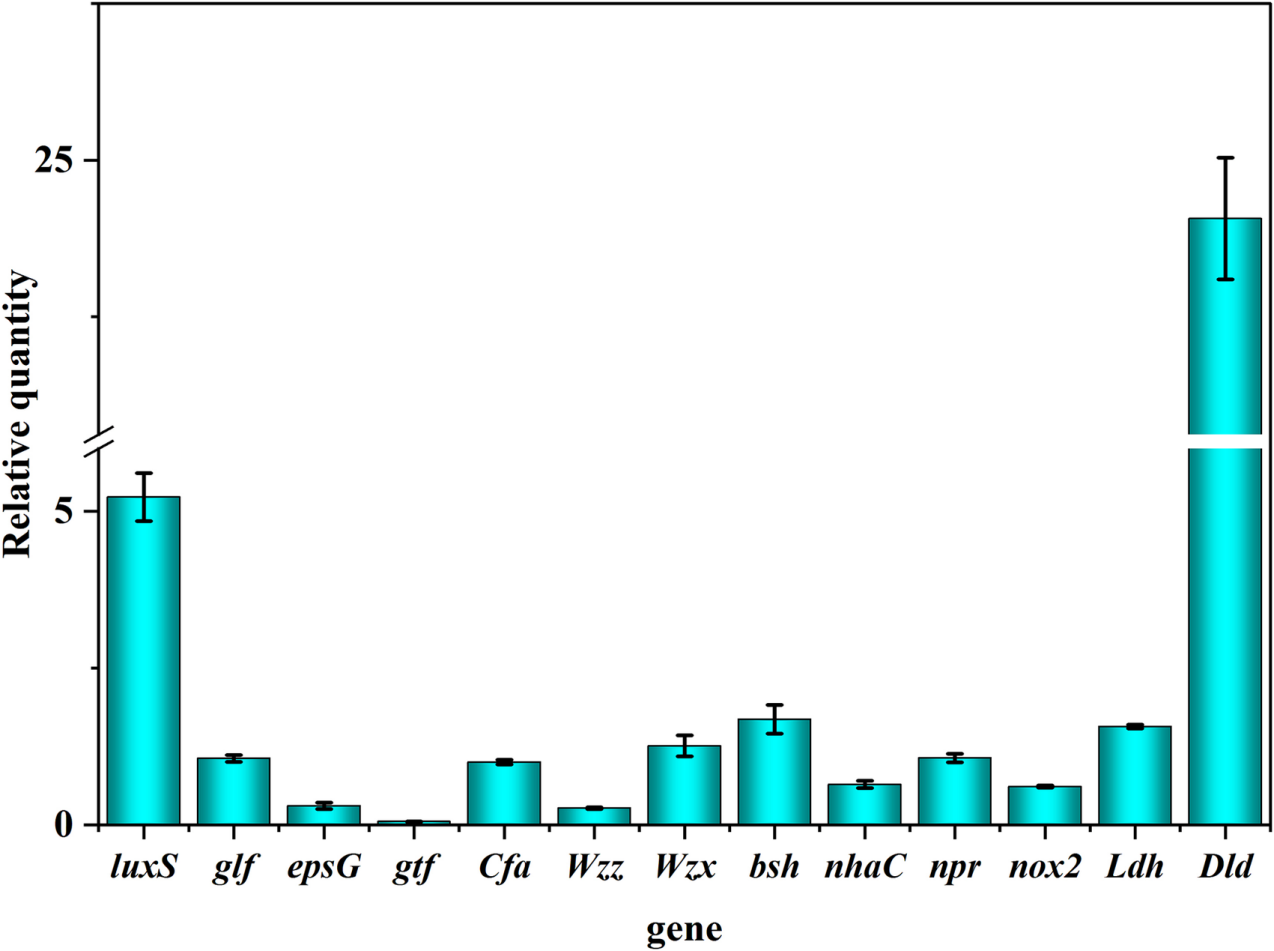
The relative expression levels of probiotic genes of *L. fermentum* A51.

**Figure 7:**
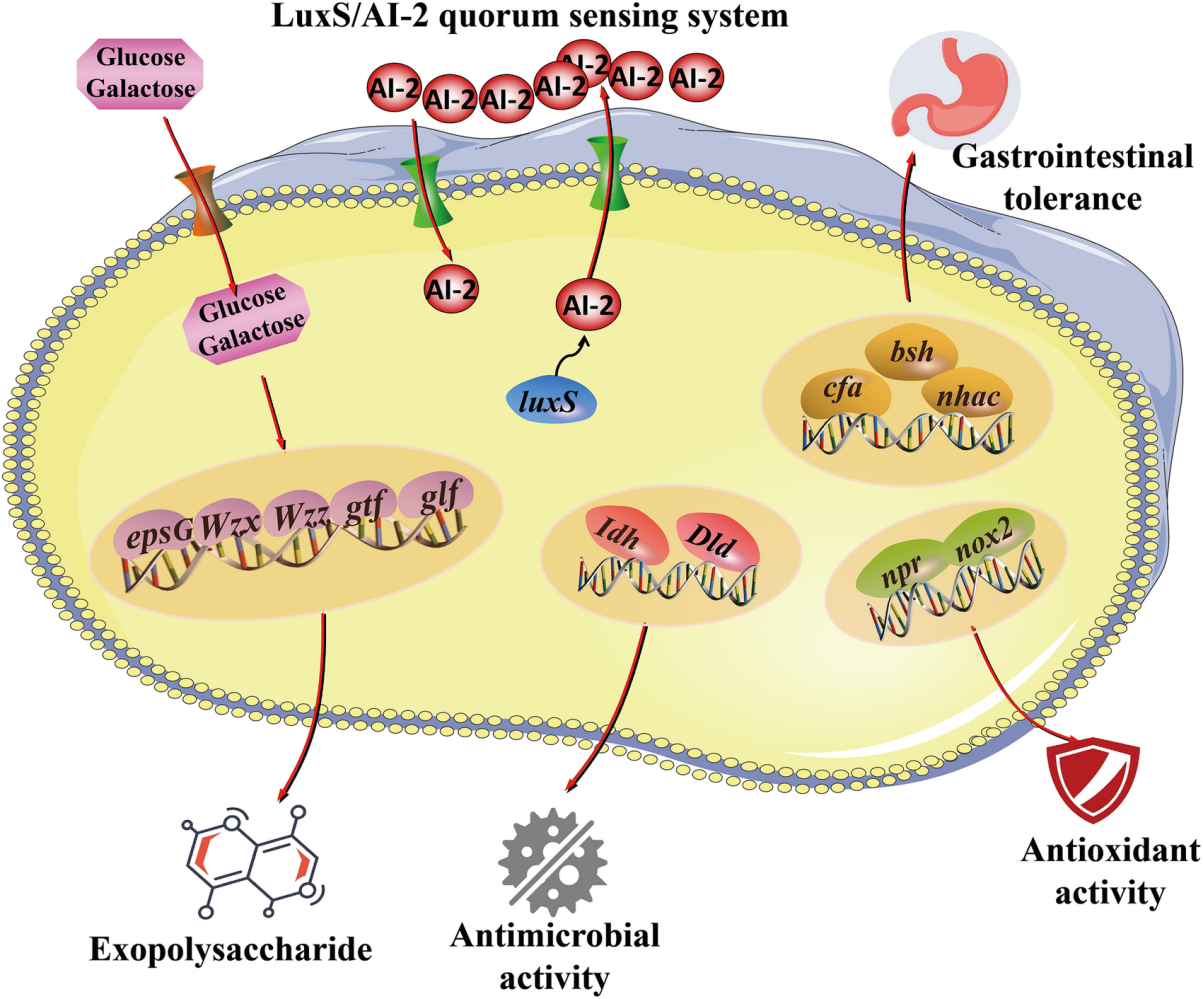
Predicted pathways for the regulation of the probiotic activities of *L. fermentum* A51.

In the current study, the expression of probiotic genes was determined, including EPS synthesis-related genes (*glf*, *epsG*, *gtf*, *Wzz,* and *Wzx*) and quorum sensing system-related genes (*luxS*), tolerance-related genes (*bsh*, *cfa,* and *nhaC*), antioxidant genes (*npr* and *nox2*) and antibacterial genes (*Idh* and *Dld*). As shown in Figure 6, genes related to probiotic properties were expressed, including EPS synthesis-related genes (*glf*), quorum sensing system genes (*luxS*), tolerance-related genes (*bsh*, *cfa,* and *nhaC*) and antimicrobial genes (*Idh* and *Dld*). These results suggest that the expression of probiotic genes contributes to the probiotic properties of *L. fermentum* A51 (Figure 7).

## 4 Conclusion

In summary, we investigated the safety and probiotic properties of *L. fermentum* A51 by whole genome mapping and phenotypic analyses, which proved that *L. fermentum* A51 is a safe strain with no antibiotic resistance genes and virulence factor genes in its genome. In addition, *L. fermentum* A51 was found to possess genes implicated in the synthesis of EPS and signaling molecule AI-2, stress response, antioxidant, and antibacterial activities, all of which led to enhanced probiotic activities, including good tolerance to simulated gastrointestinal tract, strong antioxidant and antimicrobial activities. Collectively, our findings have confirmed that *L. fermentum* A5 is safe and exhibits good probiotic properties and high exopolysaccharide production, thus recommending its potential application in the production of value-added fermented dairy products.

## Data Availability

The datasets presented in this study can be found in online repositories. The names of the repository/repositories and accession number(s) can be found at: *L. fermentum* A51 (Gene bank accession number: CP132542).

## Footnotes

^1^ https://github.com/marbl/canu

^2^ https://github.com/broadinstitute/pilon

^3^ http://blast.ncbi.nlm.nih.gov/

^4^ http://www.mgc.ac.cn/VFs/

^5^ https://card.mcmaster.ca/

## Supplementary material

SUPPLEMENTARY FIGURE S1 Evaluation of hemolysis of *L. fermentum* A51 **(A)**, *Lactobacillus casei* Zhang **(B)**, *Escherichia coli* **(C)**, and *Staphylococcus aureus* **(D)**. Positive control (*Escherichia coli* and *Staphylococcus aureus*); Negative control (*Lactobacillus casei* Zhang).

SUPPLEMENTARY TABLE S1 Gene prediction and annotation of carbohydrate transport and metabolism related genes in *L. fermentum* A51.

SUPPLEMENTARY DATA 1 The virulence factor genes of *L. fermentum* A51.

## References

[ref1] Altermann E. Russell W. M. Peril M. A. Barrangou R. Buck B. L. Mcauliffe O.. (2005). Complete genome sequence of the probiotic lactic acid bacterium *Lactobacillus acidophilus* NCFM. Proceedings of the National Academy of Sciences of the United States of America. USA 102, 3906–3912. doi: 10.1073/pnas.0409188102, PMID: 15671160 PMC554803

[ref9004] Ba M. Huang T. Guo S. Wang Y. J. Han Z. H. Li M.. (2021). Effects of extracellular polysaccharide in yogurt produced by the probiotic bacteria, lactobacillus casei zhang and bifidobacterium animalis subsp. lactis v9 on rheological properties, texture and stability. J. Chin. Inst. Food Sci. Technol. 21, 193–202. doi: 10.16429/j.1009-7848.2021.04.023

[ref2] Chai Y. M. Ma Q. W. Nong X. Mu X. Y. Huang A. X. (2023). Dissecting LuxS/AI-2 quorum sensing system-mediated phenyllactic acid production mechanisms of Lactiplantibacillus plantarum L3. Food Res. Int. 166:112582. doi: 10.1016/j.foodres.2023.112582, PMID: 36914344

[ref3] Charnchai P. Jantama S. S. Jantama K. (2017). Genome analysis of food-processing stressful-resistant probiotic *Bifidobacterium animalis* subsp. lactis BF052, and itspotential application in fermented soymilk. FEMS Microbiol. Lett. 364. doi: 10.1093/femsle/fnx180, PMID: 28911187

[ref4] Chen X. Schauder S. Potier N. Dorsselaer A. V. Pelczer I. Bassler B. L.. (2002). Structural identification of a bacterial quorum-sensing signal containing boron. Nature 415, 545–549. doi: 10.1038/415545a, PMID: 11823863

[ref5] Cheng Y. Wu T. Chu X. Tang S. Cao W. Liang F.. (2020). Fermented blueberry pomace with antioxidant properties improves fecal microbiota community structure and short chain fatty acids production in an in vitro mode. LWT 125:109260. doi: 10.1016/j.lwt.2020.109260, PMID: 39764307

[ref6] Choi E. A. Chang H. C. (2015). Cholesterol-lowering effects of a putative probiotic strain *lactobacillus plantarum* em isolated from kimchi. LWT 62, 210–217. doi: 10.1016/j.lwt.2015.01.019

[ref7] Choi K. S. Veeraragouda Y. Cho K. M. Lee S. O. Lee K. (2008). Effect of gacs and gaca mutations on colony architecture, surface motility, biofilm formation and chemical toxicity in Pseudomonas sp. kl28. J. Microbiol. 45, 492–498. doi: 10.1016/j.mimet.2007.09.016, PMID: 18176530

[ref8] Delgado S. O’Sullivan E. Fitzgerald G. Mayo B. (2007). Subtractive screening for probiotic properties of lactobacillus species from the human gastrointestinal tract in the search for new probiotics. J. Food Sci. 72, 310–315. doi: 10.1111/j.1750-3841.2007.00479.x, PMID: 17995611

[ref10] Duanis-Assaf D. Steinberg D. Chai Y. Shemesh M. (2016). The luxs based quorum sensing governs lactose induced biofilm formation by *Bacillus subtilis*. Front. Microbiol. 6, 6:1517. doi: 10.3389/fmicb.2015.01517, PMID: 26779171 PMC4705240

[ref11] Fei Y. Li L. Zheng Y. Liu D. Zhou Q. Fu L. (2018). Characterization of *Lactobacillus amylolyticus* L6 as potential probiotics based on genome sequence and corresponding phenotypes. LWT 90, 460–468. doi: 10.1016/j.lwt.2017.12.028

[ref12] Gao Y. Liu Y. Sun M. Zhang H. Tuo Y. (2020). Physiological function analysis of *Lactobacillus plantarum* y44 based on genotypic and phenotypic characteristics. J. Dairy Sci. 103, 5916–5930. doi: 10.3168/jds.2019-18047, PMID: 32418691

[ref13] Graebin N. Schöffer J. Diandra A. Plinho H. Marco A. Rafael R. (2016). Immobilization of glycoside hydrolase families GH1, GH13, and GH70: state of the art and perspectives. Molecules 21, 21:1074. doi: 10.3390/molecules21081074, PMID: 27548117 PMC6274110

[ref14] Grishina Y. V. Vatlin A. A. Mavletova D. A. Odorskaya M. V. Senkovenko A. M. Ilyasov R. A.. (2023). Metabolites potentially determine the high antioxidant properties of limosi*lactobacillus fermentum* U-21. Biotech 12:39. doi: 10.3390/BIOTECH12020039, PMID: 37218756 PMC10204573

[ref15] Gu Y. Tian J. Zhang Y. Wu R. He Y. (2020). Dissecting signal molecule AI-2 mediated biofilm formation and environmental tolerance in *lactobacillus plantarum*. J. Biosci. Bioeng. 131, 153–160. doi: 10.1016/j.jbiosc.2020.09.015, PMID: 33077360

[ref16] Gu Y. Zhang B. Tian J. Li L. He Y. (2023). Physiology, quorum sensing, and proteomics of lactic acid bacteria were affected by *Saccharomyces cerevisiae* YE4. Food Res. Int. 166:112612. doi: 10.1016/j.foodres.2023.112612, PMID: 36914328

[ref17] Iyer R. Iverson T. M. Accardi A. Miller C. (2002). A biological role for prokaryotic clc chloride channels. Nature 419, 715–718. doi: 10.1038/nature01000, PMID: 12384697

[ref18] Korcz E. Varga L. (2021). Exopolysaccharides from lactic acid bacteria: techno-functional application in the food industry. Trends Food Sci. Tech. 110, 375–384. doi: 10.1016/j.tifs.2021.02.014

[ref19] Łepecka A. Szymański P. Okoń A. Zielińska D. (2023). Antioxidant activity of environmental lactic acid bacteria strains isolated from organic raw fermented meat products. LWT 174:114440. doi: 10.1016/j.lwt.2023.114440

[ref20] Li X. Fan X. Shi Z. Xu J. Cao Y. Zhang T.. (2022). AI-2E family transporter protein in *Lactobacillus acidophilus* exhibits AI-2 exporter activity and relate with intestinal juice resistance of the strain. Front. Microbiol. 13:908145. doi: 10.3389/fmicb.2022.908145, PMID: 35633722 PMC9134010

[ref21] Li M. Li W. Li D. Tian J. Xiao L. Kwok L. Y.. (2022). Structure characterization, antioxidant capacity, rheological characteristics and expression of biosynthetic genes of exopolysaccharides produced by *Lactococcus lactis* subsp. lactis IMAU11823. Food Chem. 384:132566. doi: 10.1016/J.FOODCHEM.2022.132566, PMID: 35247774

[ref22] Li J. Y. Zhang L. J. Mu G. Q. Tuo Y. F. (2023). Interpretation of safety and potential probiotic properties of *Lactiplantibacillus plantarum* Y42 based on genome-wide sequencing. Food Biosci. 56:103249. doi: 10.1016/j.fbio.2023.103249

[ref23] Liang M.-H. Jiang J.-G. Wang L. Zhu J. (2020). Transcriptomic insights into the heat stress response of *Dunaliella bardawil*. Enzym. Microb. Technol. 132:109436. doi: 10.1016/j.enzmictec.2019.109436 31731954

[ref24] Liu D. M. Huang Y. Y. Liang M. H. (2022). Analysis of the probiotic characteristics and adaptability of lactiplantibacillus plantarum dmdl 9010 to gastrointestinal environment by complete genome sequencing and corresponding phenotypes. LWT 158:113129. doi: 10.1016/j.lwt.2022.113129, PMID: 39764307

[ref25] Liu X. Y. Xie D. Ma L. Z. Xu J. (2021). Compatative study on active xomponets and antioxidant capacity of *Rosa roxburghii* tratt.Fruit residue before and after fermentation. Food Sci. Technol. 46, 16–24.

[ref26] Lu J. Mao Y. R. Ma T. Liu X. L. Cheng X. Y.. (2023). Screening and genome analysis of lactic acid bacteria with high exopolysaccharide production and good probiotic properties. Food Biosci. 56:103211. doi: 10.1016/J.FBIO.2023.103211, PMID: 39764307

[ref27] Ma Q. W. Chai Y. M. Yang Z. B. Huang A. X. (2022). Deciphering the mechanisms of limosi*lactobacillus fermentum* L1 involved in conjugated linoleic acid regulated by LuxS/AI-2 quorum sensing-sciencedirect. LWT 154:112736. doi: 10.1016/j.lwt.2021.112736, PMID: 39764307

[ref28] Ma Y. Pan C. Wang Q. (2019). Crystal structure of bacterial cyclopropane-fatty-acyl-phospholipid synthase with phospholipid. J. Biochem. 166, 139–147. doi: 10.1093/jb/mvz018, PMID: 30828715

[ref29] Meng F. Zhao M. Lu Z. (2022). The LuxS/AI-2 system regulates the probiotic activities of lactic acid bacteria. Trends Food Sci. Tech. 127, 272–279. doi: 10.1016/j.tifs.2022.05.014

[ref30] Montoro B. P. Benomar N. Lerma L. L. Gutiérrez S. C. Gálvez A. Hikmate A. (2016). Fermented aloreña table olives as a source of potential probiotic *lactobacillus pentosus* strains. Front. Microbiol. 7, 7:1583. doi: 10.3389/fmicb.2016.01583, PMID: 27774088 PMC5054007

[ref31] Moreno-Gámez S. Sorg R. A. Domenech A. Kjos M. Weissing F. J. van Doorn G. S.. (2017). Quorum sensing integrates environmental cues, cell density and cell history to control bacterial competence. Nat. Commun. 8, 854–812. doi: 10.1038/S41467-017-00903-Y, PMID: 29021534 PMC5636887

[ref9001] Nordstrm E. A. Teixeira C. Montelius C. Jeppsson B. Larsson N. (2021). Lactiplantibacillus plantarum 299v 608(LP299V): three decades of research. Benef. Microbes. 12, 1–26. doi: 10.3920/BM2020.0191 34365915

[ref32] Oh Y. J. Kim S. A. Yang S. H. Kim D. H. Cheng Y. Y. Kang J.. (2022). Integrated genome-based assessment of safety and probiotic characteristics of *Lactiplantibacillus plantarum* PMO 08 isolated from kimchi. PLoS One 17:e0273986. doi: 10.1371/JOURNAL.PONE.0273986, PMID: 36190947 PMC9529155

[ref33] Papadimitriou K. Alegría A. Bron P. A. De Angelis M. Gobbetti M. Kleerebezem M.. (2016). Stress physiology of lactic acid bacteria. Microbiol. Mol. Biol. R. 80, 837–890. doi: 10.1128/MMBR.00076-15, PMID: 27466284 PMC4981675

[ref34] Peng Y. Y. Zhong S. Y. Xu X. L. Liu D. M. (2023). Analysis of the safety and probiotic properties of *Bifidobacterium longum* B2-01 by complete genome sequencing combined with corresponding phenotypes. LWT. 189:115445. doi: 10.1016/J.LWT.2023.115445, PMID: 39764307

[ref35] Ruas-Madiedo P. de Los Reyes-Gavilán C. G. (2005). Invited review: methods for the screening, isolation, and characterization of exopolysaccharides produced by lactic acid bacteria. J. Dairy Sci. 88, 843–856. doi: 10.3168/jds.S0022-0302(05)72750-8, PMID: 15738217

[ref36] Saadat Y. R. Khosroushahi A. Y. Gargari B. P. (2019). A comprehensive review of anticancer, immunomodulatory and health beneficial effects of the lactic acid bacteria exopolysaccharides. Carbohyd. Polym. 217, 79–89. doi: 10.1016/j.carbpol.2019.04.025, PMID: 31079688

[ref37] Salminen S. Von Wright A. Morelli L. (1998). Demonstration of safety of probiotics - a review. Inte. J. Food Microbiol. 44, 93–106. doi: 10.1016/S0168-1605(98)00128-7 9849787

[ref38] Senan S. Prajapati J. B. Joshi C. G. (2015). Whole-genome based validation of the adaptive properties of indian origin probiotic *lactobacillus helveticus* mtcc 5463. J. Sci. Food Agr. 95, 321–328. doi: 10.1002/jsfa.6721, PMID: 24798512

[ref39] Shi X. T. Greetham D. Raeth S. Perrone G. (2010). The thioredoxin-thioredoxin reductase system canfunction in vivo as an alternative system to reduce oxidized glutathione in *Saccharomyces cerevisiae*. J. Biol. Chem. 285, 6118–6126. doi: 10.1074/jbc.M109.062844, PMID: 19951944 PMC2825406

[ref40] Song J. Peng S. Yang J. Zhou F. Suo H. (2021). Isolation and identification of novel antibacterial peptides produced by *Lactobacillus fermentum* shy10 in chinese pickles. Food Chem. 348:Article 129097. doi: 10.1016/j.foodchem.2021.129097, PMID: 33515941

[ref9002] Stefanovic E. Fitzgerald G. McAuliffe O. (2017). Advances in the genomics and metabolomics of dairy lactobacilli: a review. Food Microbiol. 61, 33–49. doi: 10.1016/10.1016/j.fm.2016.08.009 27697167

[ref41] Su E. Xia T. Gao L. Dai Q. Zhang Z. (2010). Immobilization of β-glucosidase and its aroma-increasing effect on tea beverage. Food Bioprod. Process. 88, 83–89. doi: 10.1016/j.fbp.2009.04.001, PMID: 39764307

[ref42] Sun Y. Zhang S. Li H. Zhu J. Liu Z. Hu X.. (2022). Assessments of probiotic potentials of *Lactiplantibacillus plantarum* strains isolated from chinese traditional fermented food: phenotypic and genomic analysis. Front. Microbiol. 13:895132. doi: 10.3389/fmicb.2022.895132, PMID: 35615501 PMC9125032

[ref43] Trabelsi I. Slima S. B. Chaabane H. Salah R. B. (2015). Purification and characterization of a novel exopolysaccharides produced by lactobacillus sp. ca6. Int. J. Biol. Macromol. 74, 541–546. doi: 10.1016/j.ijbiomac.2014.12.045 25597428

[ref44] Vasiee A. Behbahani B. A. Yazdi F. T. Mortazavi S. A. Noorbakhsh H. (2018). Diversity and probiotic potential of lactic acid bacteria isolated from horreh, a traditional iranian fermented food. Springer 10, 258–268. doi: 10.1007/s12602-017-9282-x, PMID: 28527125

[ref45] Wang T. (2019). Effect of AI-2/LuxS quorum-sensing on S-layer protein expression and adhesion of *L. acidophilus*. Nanjing: Nanjing Normal University.

[ref46] Wang Y. J. Yang H. Y. Mu G. Q. Wu X. M. (2023). Safety evaluation and complete genome analysis emphasis on extracellular polysaccharide of two strains of *Limosilactobacillus fermentum* MWLf-4 and Lactipiantibacillus plantarum MWLp-12 from human milk. Food Biosci. 51:102356. doi: 10.1016/J.FBIO.2023.102356, PMID: 39764307

[ref47] Wei G. Q. Dai X. Y. Zhao B. Li Z. Y. Tao J. F. Wang T.. (2023). Structure-activity relationship of exopolysaccharides produced by limosi*lactobacillus fermentum* A51 and the mechanism contributing to the textural properties of yogurt. Food Hydrocoll. 144:108993. doi: 10.1016/j.foodhyd.2023.108993, PMID: 39764307

[ref48] Wu J. Han X. Ye M. Li Y. Wang X. Zhong Q. (2022). Exopolysaccharides synthesized by lactic acid bacteria: biosynthesis pathway, structure-function relationship, structural modification and applicability. Crit. Rev. Food Sci. Nutr. 63, 7043–7064. doi: 10.1080/10408398.2022.2043822, PMID: 35213280

[ref49] Wu C. C. Lin C. T. Wu C. Y. Peng W. S. Lee M. J. Tsai Y. C. (2015). Inhibitory effect of *Lactobacillus salivarius* on *Streptococcus mutans* biofilm formation. Mol Oral Microbiol 30, 16–26. doi: 10.1111/omi.12063, PMID: 24961744

[ref50] Xu X. Q. (2019). Sourdough fermented by exopolysaccharide forming strain: Application and mechanism studies in frozen dough[D]. Wuxi: Jiangnan University.

[ref51] Xue Z. P. Cu X. Xu K. Peng J. H. Liu H. R. Zhao R. T.. (2023). The effect of glutathione biosynthesis of *Streptococcus thermophilus* ST-1 on cocultured *Lactobacillus delbrueckii* ssp. bulgaricus ATCC11842. J. Dairy Sci. 106, 884–896. doi: 10.3168/JDS.2022-22123, PMID: 36460506

[ref52] Yan J. Huang Y. Gao Z. Zhang Z. Gu Q. Li P. (2023). Probiotic potential of lactiplantibacillus plantarum zfm4 isolated from pickles and its effects on human intestinal microecology. LWT 184:114954. doi: 10.1016/j.lwt.2023.114954

[ref53] Zhang Y. Du R. T. Wang L. F. Zhang H. P. (2010). The antioxidative effects of probiotic *Lactobacillus casei* zhang on the hyperlipidemic rats. Eur. Food Res. Technol. 231, 151–158. doi: 10.1007/s00217-010-1255-1

[ref54] Zhang T. Guo Y. X. Fan X. K. Liu M. Z. Xu J. Zeng X. Q.. (2023). Protection mechanism of metal ion pre-stress on *Lactobacillus acidophilus* CICC 6074 under acid tolerance. J. Agri. Food Chem 71, 13304–13315. doi: 10.1021/ACS.JAFC.3C01970, PMID: 37639527

[ref55] Zhang M. Zeng S. Hao L. Yao S. Wang D. Yang H.. (2022). Structural characterization and bioactivity of novel exopolysaccharides produced by *Tetragenococcus halophilus*. Food Res. Int. 155:111083. doi: 10.1016/j.foodres.2022.111083, PMID: 35400459

[ref56] Zhao Y. Hong K. Zhao J. Zhang H. Zhai Q. Chen W. (2019). *Lactobacillus fermentum* and its potential immunomodulatory properties. J. Funct. Foods 56, 21–32. doi: 10.1016/j.jff.2019.02.044

[ref57] Zhao Y. Yu L. Tian F. Zhao J. Zhang H. Chen W.. (2021a). An optimized culture medium to isolate *Lactobacillus fermentum* strains from the human intestinal tract. J. Funct. Foods 12, 6740–6754. doi: 10.1039/D1FO00209K, PMID: 34105590

[ref58] Zhao Y. Zhang C. C. Yu L. L. Tian F. W. Zhao J. X. Zhang H.. (2021b). Phylogenetic and comparative genomic analysis of *Lactobacillus fermentum* strains and the key genes related to their intestinal anti-inflammatory effects. Engineering 17, 170–182. doi: 10.1016/j.eng.2020.09.016, PMID: 39764307

[ref9003] Zhou Q. Gu R. Li P. Lu Y. Gu Q. (2020). Anti-salmonella mode of action of natural l-phenyl lactic acid purified from lactobacillus plantarum ZJ316. Appl. Microbiol. Biot. 104, 5283–5292. 10.1007/s00253-020-10503-4 32307571

[ref59] Zhu Z. Yang J. Yang P. Wu Z. Zhang J. Du G. (2019). Enhanced acid-stress tolerance in *Lactococcus lactis* NZ9000 by overexpression of ABC transporters. Microb. Cell Factories 18:136. doi: 10.1186/s12934-019-1188-8, PMID: 31409416 PMC6693162

