# Supplementary material for: Probiotic potential and safety properties of Limosilactobacillus fermentum A51 with high exopolysaccharide production
Source: Front Microbiol. 2025 Jan 21;16:1498352. doi: 10.3389/fmicb.2025.1498352 (PMC11790666; doi:10.3389/fmicb.2025.1498352)
Supplement: Supplementary file 1 [file Table_1.docx]

Table S1

Gene prediction and annotation of carbohydrate transport and metabolism related genes in *L. fermentum* A51.

| **Gene ID** | **Gene** | **Enzyme** | **Function annotation** |
| --- | --- | --- | --- |
| GE000424 | *gap* | EC:1.2.1.12 | glyceraldehyde 3-phosphate dehydrogenase |
| GE001366 | *pdhD* | EC:1.8.1.4 | dihydrolipoyl dehydrogenase |
| GE001367 | *pdhC* | EC:2.3.1.12 | 2-oxo acid dehydrogenase subunit E2 |
| GE001068 | *pyk* | EC:2.7.1.40 | Pyruvate kinase |
| GE000425 | *pgk* | EC:2.7.2.3 | phosphoglycerate kinase |
| GE001113 | *pck* | - | phosphoenolpyruvate carboxykinase |
| GE000427 | *enoA* | EC:4.2.1.11 | enolase |
| GE000426  GE000470 | *tpiA* | EC:5.3.1.1 | triose-phosphate isomerase |
| GE000465  GE001990 | *pgi* | EC:5.3.1.9 | glucose-6-phosphate isomerase |
| GE000402 | *pgm* | EC:5.4.2.2 | phospho-sugar mutase |
| GE000324 | *acdH* | EC:1.2.1.10 1.1.1.1 | acetaldehyde dehydrogenase |
| GE002068 | *pgd* | EC:1.1.1.44 1.1.1.343 | 6-phosphogluconate dehydrogenase |
| GE002067 | *gpd* | EC:1.1.1.49 1.1.1.363 | glucose-6-phosphate 1-dehydrogenase |
| GE002054 | *xfp, xpk* | EC:4.1.2.9 4.1.2.22 | fructose-6-phosphate phosphoketolase |
| GE001466 | *rpe* | EC:5.1.3.1 | ribulose-phosphate 3-epimerase |
| GE002027 | *rpiA* | EC:5.3.1.6 | ribose 5-phosphate isomerase |
| GE000029 | *pgmB* | EC:5.4.2.6 | phosphoglucomutase |
| GE000402 | *pgm* | EC:5.4.2.2 | phosphoglucomutase |
| GE000700 | *pgl* | EC:3.1.1.31 | 6-phosphogluconolactonase |
| GE000033  GE000788  GE001329 | *ldhA* | EC:1.1.1.28 | D-lactate dehydrogenase |
| GE001367 | *aceF, pdhC* | EC:2.3.1.12 | pyruvate dehydrogenase |
| GE002097 | *galT* | EC:2.7.7.12 | UDP-glucose--hexose-1-phosphate |
| GE002098 | *galK* | EC:2.7.1.6 | galactokinase |
| GE000345 | *galE* | EC:5.1.3.2 | UDP-glucose 4-epimerase |
| GE001963 | *galM* | EC:5.1.3.3 | aldose 1-epimerase |
| GE000402 | *pgm* | EC:5.4.2.2 | phosphoglucomutase |
| GE001501 | *glk* | EC:2.7.1.2 | glucokinase |
| GE002094 | *galA* | EC:3.2.1.22 | alpha-galactosidase |
| GE002098  GE001501 | *galK* | EC:2.7.1.6 | galactokinase |
| GE000291  GE000292 | *lacZ* | EC:3.2.1.23 | beta-galactosidase |
