# Supplementary figures and images for: Probiotic potential and safety properties of Limosilactobacillus fermentum A51 with high exopolysaccharide production
Source: Front Microbiol. 2025 Jan 21;16:1498352. doi: 10.3389/fmicb.2025.1498352 (PMC11790666; doi:10.3389/fmicb.2025.1498352)

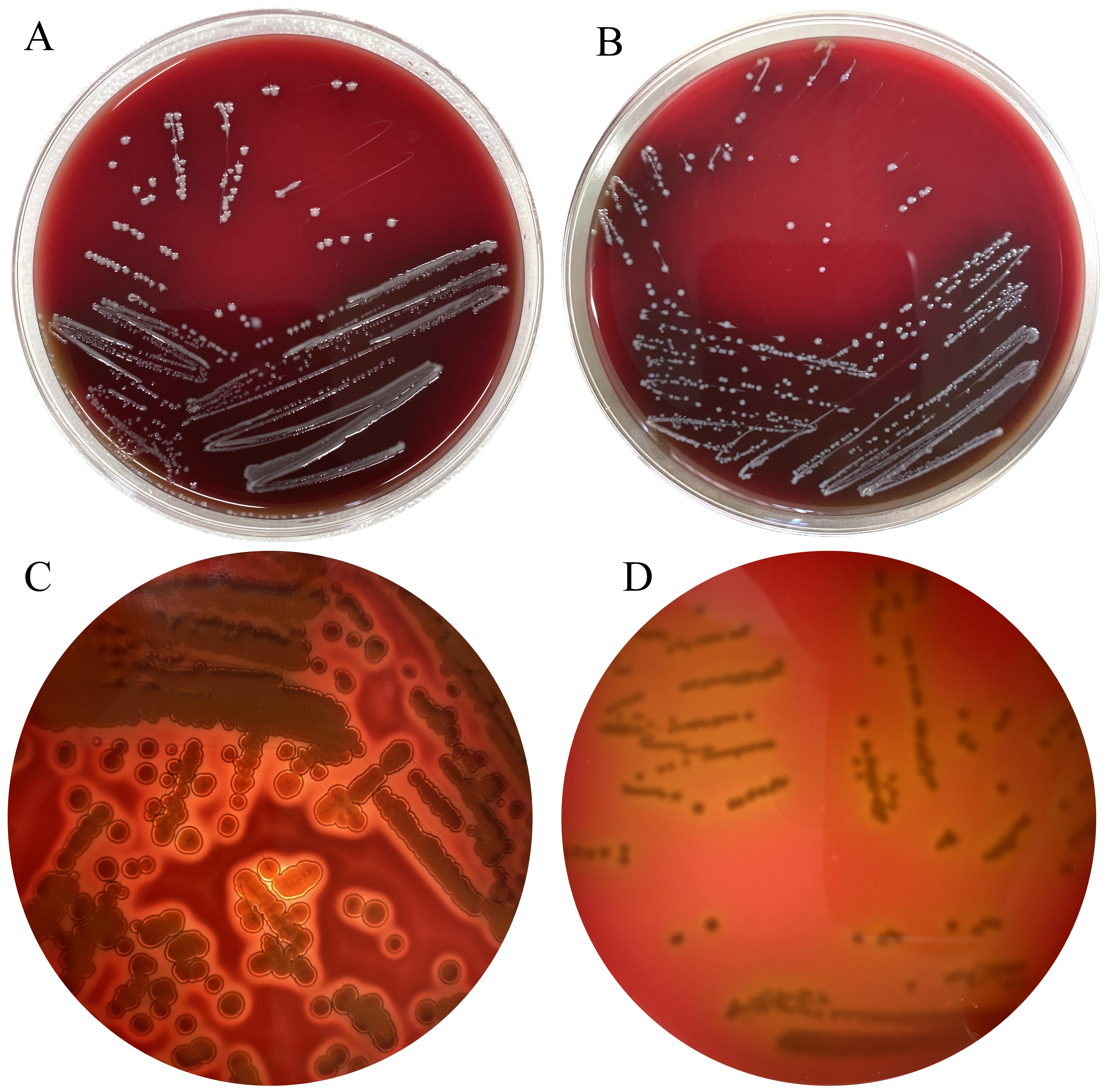

Supplement: Supplementary file 2 [file Figure_8.JPEG]

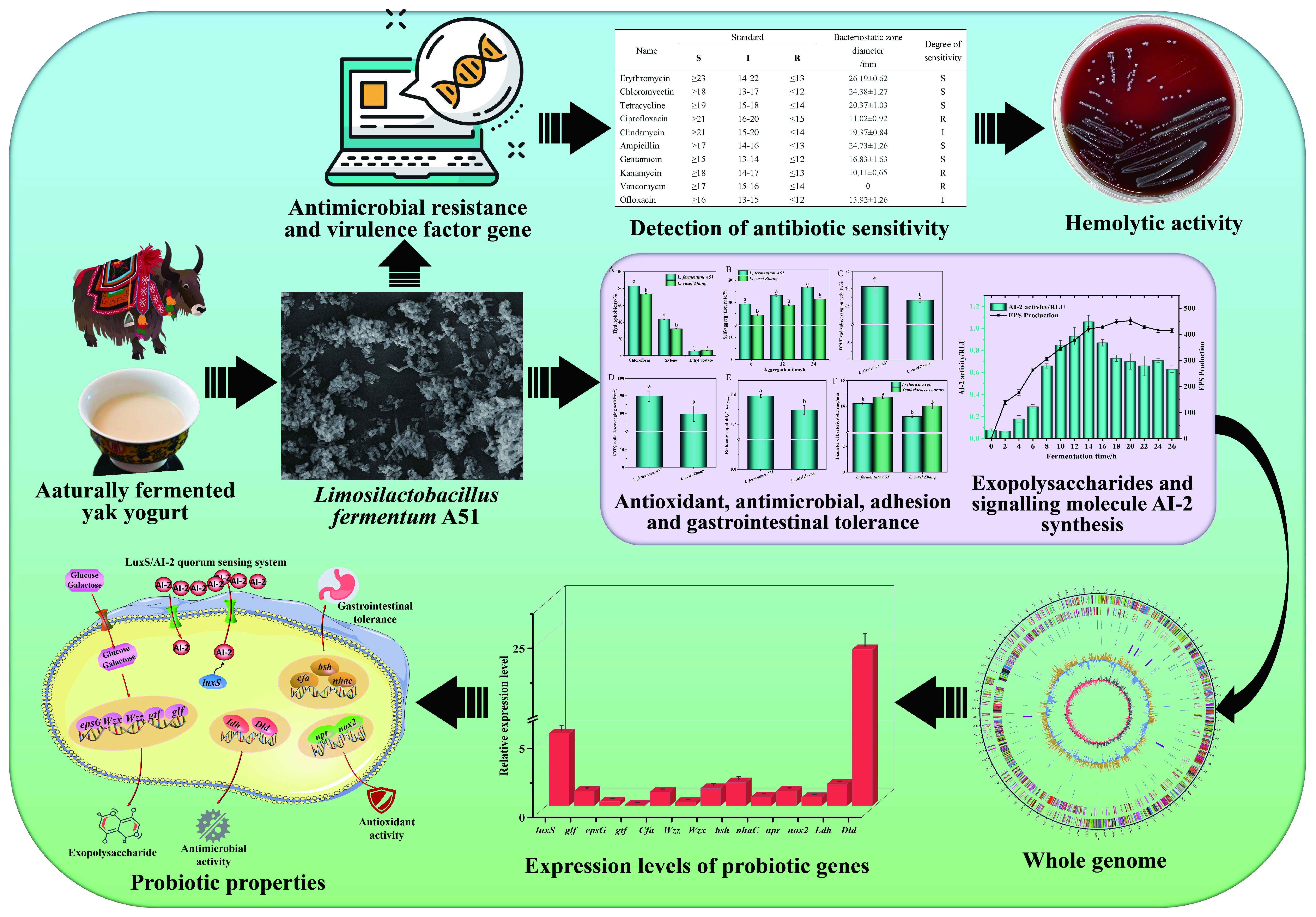

Supplement: Supplementary file 3 [file Figure_9.JPEG]
